# Signal-to-Noise Ratio in Estimating and Testing the Mediation Effect: Structural Equation Modeling versus Path Analysis with Weighted Composites

**DOI:** 10.1007/s11336-024-09975-4

**Published:** 2024-05-28

**Authors:** Ke-Hai Yuan, Zhiyong Zhang, Lijuan Wang

**Affiliations:** 1 Renmin University of China, Beijing, China; 2 University of Notre Dame, Notre Dame, Indiana 46556 USA

**Keywords:** factor-score regression, structural equation modeling, indirect effect, measurement reliability, signal-to-noise ratio

## Abstract

Mediation analysis plays an important role in understanding causal processes in social and behavioral sciences. While path analysis with composite scores was criticized to yield biased parameter estimates when variables contain measurement errors, recent literature has pointed out that the population values of parameters of latent-variable models are determined by the subjectively assigned scales of the latent variables. Thus, conclusions in existing studies comparing structural equation modeling (SEM) and path analysis with weighted composites (PAWC) on the accuracy and precision of the estimates of the indirect effect in mediation analysis have little validity. Instead of comparing the size on estimates of the indirect effect between SEM and PAWC, this article compares parameter estimates by signal-to-noise ratio (SNR), which does not depend on the metrics of the latent variables once the anchors of the latent variables are determined. Results show that PAWC yields greater SNR than SEM in estimating and testing the indirect effect even when measurement errors exist. In particular, path analysis via factor scores almost always yields greater SNRs than SEM. Mediation analysis with equally weighted composites (EWCs) also more likely yields greater SNRs than SEM. Consequently, PAWC is statistically more efficient and more powerful than SEM in conducting mediation analysis in empirical research. The article also further studies conditions that cause SEM to have smaller SNRs, and results indicate that the advantage of PAWC becomes more obvious when there is a strong relationship between the predictor and the mediator, whereas the size of the prediction error in the mediator adversely affects the performance of the PAWC methodology. Results of a real-data example also support the conclusions.

## Introduction

Mediation analysis facilitates the understanding of the process of the causal effect of a predictor on the outcome variable and plays an important role in studying the effect of intervention (see, e.g., Hayes, 2018; MacKinnon, 2008; Zhang & Yang, 2020). Because data in social and behavioral sciences are often observational and typically contain measurement errors, structural equation modeling (SEM) has been believed a better method for conducting mediation analysis than path analysis^1^ with weighted composites (PAWC). In this article, we show that this belief is not true in general. Instead of comparing the two classes of methods by the size of their parameter estimates, we propose to use signal-to-noise ratio (SNR) to measure the efficiency of the estimates of the indirect effect and show that PAWC performs better than SEM under the new measure. We will formally define the concept of SNR in the following section and show that it is directly related to the statistical power in detecting the existence of an indirect effect. Because statistical power is a top concern in mediation analysis, via the analysis of the results under many conditions, we will also identify key factors that cause the differences of the SNRs between different methods.

Measurement errors cannot be avoided with observational data. When predictors contain measurement errors, regression analysis via composites tends to yield estimates that are inconsistent with those among latent variables. By explicitly modeling measurement errors, the method of SEM can consistently estimate the values of the coefficients that govern the relationship among the latent constructs as well as other parameters of the model. However, model misspecification also cannot be avoided in practice (e.g., Box, 1979; MacCallum, 2003), which will result in biased representations of the theoretical constructs by the latent variables as well as parameter estimates that are systematically different from their counterparts under a correctly specified model (Yuan et al. 2003). Thus, we regard both latent variables and weighted composites as approximations to the theoretical constructs. But we do not explicitly consider the issue of model misspecification in this article, and the setup implicitly favors the SEM methodology.

Croon (2002) discussed issues in statistical modeling when latent variables are replaced by factor scores or composites. A consequence of the replacement is that the path coefficients under PAWC are systematically different from their counterpart under latent-variable models, as has been noted in textbooks (Allen and Yen, 1979; McDonald, 1999). However, because latent variables in social and behavioral sciences typically do not carry units, we have to assign a scale to each latent variable for an SEM model to be identified. For an exogenous latent variable, this can be done by fixing the factor loading of one of its indicators at 1.0 or by fixing the variance of the latent variable at 1.0. But the choice between the two is arbitrary (e.g., Bentler, 2006). The scale of an endogenous latent variable is typically aligned to one of its indicators by fixing the factor loading at 1.0. Still, the choice of the indicator as well as the value of 1.0 are arbitrary. Each of the listed arbitrarinesses makes the values of the path coefficients among the latent variables artificial (Yuan and Deng, 2021; Yuan and Zhang, 2023). Similarly, the scales of weighted composites can also be chosen artificially (e.g., sum score versus average score), and the values of the corresponding regression coefficients depend on the chosen scales. In addition, for the value of a path coefficient defined under a latent-variable model, one can obtain an identical value under regression analysis using weighted composites by adjusting the scales of the composites, and vice versa (see, e.g., Hoshino & Bentler, 2013; Skrondal & Laake 2001; Yuan & Deng, 2021; Yuan & Zhang, 2023). Consequently, the differences in parameter estimates between SEM and PAWC are artificial rather than substantively grounded. Such an observation leaves more space for researchers to select a method according to a specific purpose. In particular, we should choose a method that has the greatest statistical power if the interest is to detect the existence of a mediation relationship among the theoretical constructs. Our study in comparing the SNRs for the estimates of the indirect effect is a clarification of the issues discussed by Croon (2002), especially for mediation analysis in social and behavioral sciences where measurements typically contain errors and do not have predefined metrics.

For mediation analysis, Ledgerwood and Shrout (2011) compared SEM and path analysis via average item scores with respect to bias and standard errors (SEs) of parameter estimates. They used “accuracy” and “precision” to substitute for bias and SEs, and their Monte Carlo results showed that SEM yields estimates with greater accuracy but less precision. While Ledgerwood and Shrout’s findings are interesting, they have little validity. This is because both the values of parameter estimates and their SEs under both SEM and PAWC depend on the subjectively chosen scales of the involved variables, as noted above. In addition, Ledgerwood and Shrout (2011) only considered indicators whose true-score variances are equal and so are the error variances, termed as parallel tests or measurements (Allen and Yen, 1979). Because, with parallel indicators, the average scores attain maximum reliability (Bentler, 1968; Yuan & Bentler, 2002) and the estimates of the path coefficients and their SEs depend on the reliabilities of the composites (Cochran, 1970; Fuller, 1987), the results of Ledgerwood and Shrout (2011) are of little generalizability. For the same reason, direct parameter comparison between SEM and PAWC for moderated-mediation analysis is not well-grounded either (Cheung and Lau, 2017).

There has been a great interest in determining the existence of a mediation effect. This is typically done by a null-hypothesis statistical test (NHST) for the indirect effect of the predictor on the outcome variable via the mediator, or by using the confidence interval (CI) approach to inference (MacKinnon et al., 2002; Shrout and Bolger, 2002). Other approaches of quantifying mediation effects also exist (e.g., Fairchild et al., 2009; Lachowicz, Preacher & Kelley, 2018; Liu et al., 2024; Preacher & Kelley, 2011). The focus of this article is on the SNR for estimating the indirect effect. Since the power of NHST is directly related to SNR, our aim is to identify the methods that yield the greatest SNRs. We will not study type I errors because they can be properly controlled via the bootstrap or Monte Carlo methods (see, e.g., Efron & Tibshirani, 1993; Hayes & Scharkow, 2013; Miocevic et al., 2018; Yuan & Hayashi, 2003; Yuan, Zhang & Zhao, 2017). Readers interested in bootstrap methods for type I error control in mediation analysis are referred to MacKinnon et al. (2002) and Tibbe and Montoya (2022). Because the bootstrap methods are nonparametric, the validity of their findings is expected to hold regardless of the size of the SNRs for estimators of the indirect effect. We will discuss how SNR is related to the width of confidence intervals and statistical power in the following section.

Our interest in SNR for mediation analysis by different methods is motivated by a recent study by Yuan and Fang (2023) who showed that, conditional on the weights, the SNR for the regression coefficient between two latent variables under SEM is mathematically smaller than that under regression analysis via the Bartlett- or regression-factor scores. When sampling errors in weights are considered, the average SNR for the estimated regression coefficient using the Bartlett-factor scores becomes even greater. Since SNR plays a pivotal role in NHST, we are interested in whether the finding in Yuan and Fang (2023) still holds for the indirect effect in mediation analysis. It turns out that the mathematics in analytically studying the SNR for the indirect effect is much more complicated than that for the regression coefficient between two latent variables, due to the confounding effects with correlated variables. So we will use a mixed approach by first driving the asymptotic covariance matrix of parameter estimates and then numerically compare the resulting SNRs by different methods. Different SNRs will also be compared empirically via Monte Carlo simulation.

In sum, the purpose of this article is to rigorously compare PAWC against SEM with respect to the SNRs of the indirect effect in mediation analysis. Our contributions include: (1) Noting that it is not meaningful to compare the sizes of parameters between SEM and PAWC for mediation analysis with theoretical constructs; (2) proposing to use the size of SNR to measure the efficiency of different methods in conducting mediation analysis; (3) rigorously studying the performances of SEM and PAWC in estimating and testing the indirect effect with respect to SNR; (4) identifying the causes for the SNR difference between SEM and PAWC for the indirect effect in mediation analysis; and (5) developing a formula to account for the effect of the estimated weights in the Bartlett-factor scores for computing the standard errors of the indirect effect in mediation analysis. We will conduct the study by combining analytical results with numerical analysis. Analytical results are presented in the following section, and numerical results are presented in a subsequent section. Factors that contribute to the advantage and disadvantage of PAWC are examined in a follow-up section. A real-data example comparing the different approaches are provided in a separate section. Discussion and recommendations are offered in the concluding section. Technical details of the analytical results are given in appendices.

## Analytical Formulas of Signal-to-Noise Ratios

In this section, we will first define the concept of SNR (signal-to-noise ratio) and then present its formulation and computation for the indirect effects under SEM and PAWC. The main task is to relate the SNRs under PAWC to parameters under SEM. We will only consider the condition with normally distributed data since the widely used normal-distribution-based maximum likelihood (NML) method for SEM is known to yield most efficient parameter estimates, which correspond to the greatest SNR. If SEM does not have an obvious advantage over PAWC under such a condition, then SEM may be even less desirable for mediation analysis when data are not normally distributed. We will further discuss this issue in the concluding section.

There are two scenarios for computing the SNRs under PAWC. The first one is when the weights in the formulations of the composites are considered as given or the analysis is conditional on a particular set of estimated weights. Examples of this type include treating factor scores as observed data (see, e.g., DiStefano et al., 2009) in the analysis or when the sum score or equally weighted composites (EWCs) are used. It also includes cases when items are weighted according to design or theoretical consideration. The second scenario accounts for the sampling errors in the estimated weights, which includes cases when factor scores are computed from sample to sample as in Monte Carlo study or analysis via the bootstrap methodology.

### Signal-to-Noise Ratio (SNR)

For a parameter estimate ${\hat{\theta }}$ based on a sample of size *N*, let $\theta$ be the expected or asymptotic value of ${\hat{\theta }}$, and $\textrm{SD}_{\theta }=[\textrm{Var}(\sqrt{N}{\hat{\theta }})]^{1/2}$ be the square root of the variance of $\sqrt{N}{\hat{\theta }}$, termed as the standard deviation of ${\hat{\theta }}$ in this article. We define the SNR of ${\hat{\theta }}$ as

1 $$\begin{aligned} \tau =\theta /\textrm{SD}_{\theta }. \end{aligned}$$

Note that the SNR in (1) depends on the population distribution of the sample based on which ${\hat{\theta }}$ is computed in addition to the estimation method used to obtain ${\hat{\theta }}$. However, SNR does not depend on the scales of the involved variables for all sensible estimation methods. For example, if $\theta =\mu$ is the mean of a univariate population represented by *x* and ${\hat{\theta }}=\bar{x}$ is the sample mean, then $\tau =\mu /\sigma$ does not depend on the scale of *x*, where $\sigma$ is the population standard deviation of the random variable *x*. Similarly, if $\theta$ is the regression coefficient of *y* on *x* and ${\hat{\theta }}$ is obtained by least squares or a robust method, then the corresponding $\tau$ does not depend on the metric of either *x* or *y*. For latent variables in an SEM model whose scales are fixed by letting one of their loadings at 1.0 or by setting their variances at 1.0, then $\tau$ remains the same for all parameter estimates when the chosen loadings or variances are reset at any other positive values. Also, $\tau$ (asymptotically) does not depend on the sample size *N* or (with finite samples) depends little on the sample size *N*. This is because $\textrm{SE}_{\theta }=[\textrm{Var}({\hat{\theta }})]^{1/2}$ is inversely proportional to $N^{1/2}$ for essentially all parameter estimates (Casella and Berger, 2002; Ferguson, 1996), and $\textrm{SD}_{\theta }=\sqrt{N}\textrm{SE}_{\theta }$ effectively removes the dependence on *N*.

While the SNR is a stand-alone concept, it can be regarded as a generalization to $\tau =\mu /\sigma$ for the sample mean ${\hat{\theta }}=\bar{x}$ and applies to any parameter estimate. The concept SNR facilitates comparison of the goodness of parameter estimates by different methods as well as comparison of findings by different researchers who might have scaled variables differently in their studies.

Since $\tau$ is the expected value of ${\hat{\theta }}$ over its $\textrm{SD}$, an estimator with a greater SNR has a smaller relative error. Consequently, statistical testing based on a parameter estimate with a greater SNR corresponds to a sampling distribution with a greater non-centrality parameter (NCP) when the hypothesis $H_0$: $\theta =0$ does not hold. Among estimates of $\theta$ by different methods, the $\alpha$-level confidence interval (CI) for $\theta$ based on an estimate with a greater SNR will be shorter if the estimates are made equal by rescaling the related variables. Thus, test statistics or inference via CI based on parameter estimates with greater SNRs will be statistically more powerful.

When $\theta =\mu$ is the population mean of a univariate sample, the corresponding SNR can be estimated by ${\hat{\tau }}=\bar{x}/s=t/\sqrt{N}$, where $t=\sqrt{N}\bar{x}/s$ is the student *t*-statistic for testing $H_0$: $\mu =0$. Let the *z*-statistic for ${\hat{\theta }}$ be defined as $z={\hat{\theta }}/\textrm{SE}$, then the corresponding $\tau$ can be estimated as ${\hat{\tau }}=z/\sqrt{N}$. However, the SNR intends to serve as a measure of the goodness of an estimator rather than serving as a test statistic. In particular, the SNR is a population quantity that does not depend on sample size. In contrast, the p-value, power of a test statistic, and the NCP for testing $H_0$: $\theta =0$ typically depend on sample size.

Note that the SNR in Eq. (1) is a standardized difference between $\theta$ and 0. A multivariate version of SNR was given in Yuan and Fang (2023), which is essentially the Mahalanobis distance between vectors ${\varvec{\theta }}$ and $\textbf{0}$ weighted by the precision matrix $[\textrm{Cov}(\sqrt{N}\hat{{\varvec{\theta }}})]^{-1}$.

SEM and path analysis with differently weighted composites represent different estimation methods, and consequently, they correspond to different SNRs. The following sections will be around comparing the SNRs of parameter estimates by these methods for mediation analyses, with the indirect effect being the focal parameter.

### Indirect Effect Under SEM

Let $\textbf{x}$, $\textbf{y}_1$ and $\textbf{y}_2$ be vectors of mean-centered indicators, and we have the following latent-variable model for studying the indirect effect

2 $$\begin{aligned}{} & {} \textbf{x}={\varvec{\lambda }}_x\xi +\textbf{e}_x, \;\; \textbf{y}_1={\varvec{\lambda }}_{y_1}\eta _1+\textbf{e}_{y_1}, \;\; \textbf{y}_2={\varvec{\lambda }}_{y_2}\eta _2+\textbf{e}_{y_2}, \end{aligned}$$

3 $$\begin{aligned}{} & {} \eta _1=\gamma _1\xi +\zeta _1, \;\; \eta _2=\beta _1\eta _1+\gamma _2\xi +\zeta _2, \end{aligned}$$

where ${\varvec{\lambda }}_x$, ${\varvec{\lambda }}_{y_1}$ and ${\varvec{\lambda }}_{y_2}$ are vectors of factor loadings; $\textbf{e}_x$, $\textbf{e}_{y_1}$, and $\textbf{e}_{y_2}$ are vectors of errors that are independent from each other and from the latent variables $\xi$, $\eta _1$ and $\eta _2$; and the residuals $\zeta _1$ and $\zeta _2$ are independent with the predictors in the same equation. With mean-centered indicators, all the latent variables, errors and residuals have mean zero; $\textrm{Cov}(\textbf{e}_x)={\varvec{\Psi }}_x$, $\textrm{Cov}(\textbf{e}_{y_1})={\varvec{\Psi }}_{y_1}$ and $\textrm{Cov}(\textbf{e}_{y_2})={\varvec{\Psi }}_{y_2}$ are diagonal matrices; $\textrm{Var}(\zeta _1)=\sigma _{\zeta _1}^2$ and $\textrm{Var}(\zeta _2)=\sigma _{\zeta _2}^2$. In the context of mediation analysis under SEM, $\eta _1$ is a latent mediator, and the interest is the indirect effect of $\xi$ on $\eta _2$ via $\eta _1$, quantified by the product $\gamma _1\beta _1$.

Suppose there are $p_x$, $p_{y_1}$ and $p_{y_2}$ indicators in $\textbf{x}$, $\textbf{y}_1$ and $\textbf{y}_2$, respectively. We let $\sigma _{\xi }^2=\textrm{Var}(\xi )=1.0$ and the first loadings in ${\varvec{\lambda }}_{y_1}$ and ${\varvec{\lambda }}_{y_2}$ be 1.0 for the purpose of model identification. Then, there are $q=2(p_x+p_{y_1}+p_{y_2})+3$ model parameters in Eqs. (2) and (3). Let ${\varvec{\theta }}$ denote the vector of the *q* parameters, and we will refer to ${\varvec{\theta }}$ as the base parameters. With normally distributed data, ${\varvec{\theta }}$ can be efficiently estimated by NML. Let ${\hat{\gamma }}_1$ and ${\hat{\beta }}_1$ be the NML estimates of $\gamma _1$ and $\beta _1$, respectively. The indirect effect is then estimated as ${\hat{\gamma }}_1{\hat{\beta }}_1$, whose population counterpart is $\gamma _1\beta _1$.

We need the SD of the NML estimate ${\hat{\gamma }}_1{\hat{\beta }}_1$ in order to compute the corresponding SNR according to Eq. (1). For the *q* estimates in $\hat{{\varvec{\theta }}}$, their covariance matrix is consistently estimated by the inverse of the information matrix (i.e., the matrix of the expected values of the 2nd derivatives of the log likelihood function multiplied by $-1$). The diagonal elements of this covariance matrix are used to compute the default standard errors (SEs) of $\hat{{\varvec{\theta }}}$ in SEM software (e.g., Bentler, 2006; Rosseel, 2012). Let

$$\begin{aligned} \textbf{V}=\left( \begin{array}{cc} v_{gg}&{}\;\; v_{gb}\\ v_{bg}&{}\;\; v_{bb} \end{array} \right) \end{aligned}$$

be the covariance matrix of $({\hat{\gamma }}_1,{\hat{\beta }}_1)'$, which is just a sub-matrix of the covariance matrix of $\hat{{\varvec{\theta }}}$. Then, the asymptotic SE for the NML estimate ${\hat{\gamma }}_1{\hat{\beta }}_1$ of the indirect effect can be obtained by the so-called delta-method (Ferguson, 1996), which is given by

4 $$\begin{aligned} \textrm{SE}_{\gamma _1\beta _1}=(\beta _1^2 v_{gg}+\gamma _1^2 v_{bb}+2\beta _1\gamma _1v_{gb})^{1/2}. \end{aligned}$$

Note that ${\hat{\gamma }}_1$ and ${\hat{\beta }}_1$ are correlated in general (i.e., $v_{gb}\ne 0$) even when $\textbf{x}$, $\textbf{y}_1$ and $\textbf{y}_2$ are all normally distributed; and $v_{gg}$, $v_{bb}$ and $v_{gb}$ are functions of ${\varvec{\theta }}$, not just functions of $\gamma _1$ and $\beta _1$. With Eq. (4), the SD of ${\hat{\gamma }}_1{\hat{\beta }}_1$ is given by:

5 $$\begin{aligned} \omega _{\gamma _1\beta _1}=\sqrt{N}\textrm{SE}_{\gamma _1\beta _1}, \end{aligned}$$

which is a function of the base parameters in ${\varvec{\theta }}$ alone (unrelated to *N*) and can be evaluated via Eqs. (4) and (5). Thus, with normally distributed data, the SNR corresponding to the indirect effect under SEM is defined as:

6 $$\begin{aligned} \tau _{\gamma _1\beta _1}=\frac{\gamma _1\beta _1}{\omega _{\gamma _1\beta _1}}. \end{aligned}$$

We will refer to $\tau _{\gamma _1\beta _1}$ as the population or asymptotic SNR under SEM in the following presentation.

Another SNR for the indirect effect might be defined via Eq. (3) alone, with $\tau _0=\gamma _1\beta _1/\omega _0$, where $\omega _0$ is the standard deviation of ${\tilde{\gamma }}_1{\tilde{\beta }}_1$ corresponding to path analysis when the latent variables $\xi$, $\eta _1$ and $\eta _2$ are literally available (e.g., in Monte Carlo studies). However, this SNR has little to do with testing the significance of ${\hat{\gamma }}_1{\hat{\beta }}_1$ under SEM. This is because in practice the relationship among latent variables can only be estimated by including the measurement model in Eq. (2). Even in the extreme case when the numbers of indicators ($p_x$, $p_{y_1}$ and $p_{y_2}$) for all the latent variables become infinitely large, the SDs of the NML estimates ${\hat{\gamma }}_1$ and ${\hat{\beta }}_1$ are still greater than those when latent variables are literally obtainable (Yuan and Fang, 2023). The reason for this is that the number of factor loadings and error variances under SEM proportionally increases with $p_x$, $p_{y_1}$ and $p_{y_2}$. As the number of indicators increases, the information gained is mostly used by estimating these model parameters.

### Indirect Effect Under PAWC Conditional on Weights

We derive the formula for computing the SNR of the indirect effect under PAWC in this subsection, where weights are held constant. Specific forms of composites will be considered in the next section when the SNRs are numerically compared. Let $\textbf{w}_x$, $\textbf{w}_{y_1}$ and $\textbf{w}_{y_2}$ be vectors of weights corresponding to the indicators $\textbf{x}$, $\textbf{y}_1$ and $\textbf{y}_2$, respectively. Corresponding to the model in Eqs. (2) and (3), let ${\hat{\xi }}=\textbf{w}_x'\textbf{x}/(\textbf{w}_x'{\varvec{\lambda }}_x)$, ${\hat{\eta }}_1=\textbf{w}_{y_1}'\textbf{y}_1/(\textbf{w}_{y_1}'{\varvec{\lambda }}_{y_1})$ and ${\hat{\eta }}_2=\textbf{w}_{y_2}'\textbf{y}_2/(\textbf{w}_{y_2}'{\varvec{\lambda }}_{y_2})$ be the weighted composites, where the role of $\textbf{w}'{\varvec{\lambda }}$ in the denominators is to make the resulting composites to have a 1-1 link with the latent variables (i.e., ${\hat{\xi }}=\xi +e_{{\hat{\xi }}}$, ${\hat{\eta }}_1=\eta _1+e_{{\hat{\eta }}_1}$, and ${\hat{\eta }}_2=\eta _2+e_{{\hat{\eta }}_2}$). Such a rescaling does not affect the SNR for the indirect effect under PAWC. Note that hats are used to distinguish the weighted composites from the error-free latent variables and, for the purpose of studying the population SNR, the weights are still at the population level instead of empirically estimated. For the weighted composites, we have the following models for mediation analysis

7 $$\begin{aligned} {\hat{\eta }}_1=a{\hat{\xi }}+e_1, \;\; {\hat{\eta }}_2=b{\hat{\eta }}_1+c{\hat{\xi }}+e_2, \end{aligned}$$

and the product *ab* is commonly called the indirect or mediation effect. However, the value of *ab* depends on the scales of the weighted composites the same way as $\gamma _1\beta _1$ depends on the scales of the latent variables. The SNR will remove such dependency by accounting for the sampling errors in the estimate $\hat{a}\hat{b}$.

Because ${\hat{\xi }}$, ${\hat{\eta }}_1$ and ${\hat{\eta }}_2$ are derived from Eqs. (2) and (3), we need to relate the value of *ab* to the base parameters in ${\varvec{\theta }}$. It follows from the forms of the composites that their variances are given by

8 $$\begin{aligned} \sigma _{{\hat{\xi }}}^2=\textrm{Var}({\hat{\xi }})=\sigma _{\xi }^2/\rho _{{\hat{\xi }}}, \;\; \sigma _{{\hat{\eta }}_1}^2=\textrm{Var}({\hat{\eta }}_1)=\sigma _{\eta _1}^2/\rho _{{\hat{\eta }}_1}, \;\;\textrm{and}\;\; \sigma _{{\hat{\eta }}_2}^2=\textrm{Var}({\hat{\eta }}_2)=\sigma _{\eta _2}^2/\rho _{{\hat{\eta }}_2}, \end{aligned}$$

where $\sigma _{\xi }^2$, $\sigma _{\eta _1}^2$ and $\sigma _{\eta _2}^2$ are the variances of $\xi$, $\eta _1$ and $\eta _2$; and $\rho _{{\hat{\xi }}}$, $\rho _{{\hat{\eta }}_1}$ and $\rho _{{\hat{\eta }}_2}$ are the reliability coefficients of ${\hat{\xi }}$, ${\hat{\eta }}_1$ and ${\hat{\eta }}_2$, respectively. Because the functional forms of these coefficients are not essential in understanding the development, they are given in Appendix A presented at the end of the article. Note that we used $\sigma _{\xi }^2=\textrm{Var}(\xi )$ instead of replacing its value by 1.0 for the purpose of clarity. Because measurement errors are independent with the latent constructs, the covariances among the composites are given by:

9 $$\begin{aligned} \begin{array}{c} \sigma _{{\hat{\xi {\hat{\eta }}}}_1}=\textrm{Cov}({\hat{\xi }},{\hat{\eta }}_1)=\gamma _1\sigma _{\xi }^2, \;\; \sigma _{{\hat{\xi {\hat{\eta }}}}_2}=\textrm{Cov}({\hat{\xi }},{\hat{\eta }}_2) =(\beta _1\gamma _1+\gamma _2)\sigma _{\xi }^2,\\ \sigma _{{\hat{\eta }}_1{\hat{\eta }}_2}=\textrm{Cov}({\hat{\eta }}_1,{\hat{\eta }}_2) =\beta _1\sigma _{\eta _1}^2+\gamma _1\gamma _2\sigma _{\xi }^2. \end{array} \end{aligned}$$

Thus, the population value of *a* in Eq. (7) is given by

10 $$\begin{aligned} a=\sigma _{{\hat{\xi {\hat{\eta }}}}_1}/\sigma _{{\hat{\xi }}}^2=\rho _{{\hat{\xi }}}\gamma _1. \end{aligned}$$

It is obvious that $a=\gamma _1$ when $\rho _{{\hat{\xi }}}=1$. However, $a<\gamma _1$ in general. Yuan and Fang (2023) showed that the SNR of $\hat{a}$ by LS regression is always greater than its SEM counterpart when factor scores are used in the regression model, and we will also numerically illustrate this in the next section.

The method of LS regression allows us to obtain the population values of *b* and *c* using the standard formula ${\varvec{\beta }}={\varvec{\Sigma }}_{11}^{-1}{\varvec{\sigma }}_{12}$, where ${\varvec{\Sigma }}_{11}$ is the covariance matrix of the involved predictors and ${\varvec{\sigma }}_{12}$ is the vector of covariances between the predictors and the dependent variable. With the variances and covariances in Eqs. (8) and (9), together with the formula for inverting a $2\times 2$ matrix in Eq. (B5) of Appendix B, we have

11 $$\begin{aligned} \left( \begin{array}{c} b\\ c \end{array} \right) = \frac{1}{\sigma _{\zeta _1}^2/(\rho _{{\hat{\xi }}}\rho _{{\hat{\eta }}_1})+ \gamma _1^2\sigma _{\xi }^2[1/(\rho _{{\hat{\xi }}}\rho _{{\hat{\eta }}_1})-1] } \left( \begin{array}{c} \gamma _1(\beta _1\gamma _1+\gamma _2)\sigma _{\xi }^2(1/\rho _{{\hat{\xi }}}-1)+\beta _1\sigma _{\zeta _1}^2/\rho _{{\hat{\xi }}}\\ \gamma _1(\beta _1\sigma _{\eta _1}^2+\gamma _1\gamma _2\sigma _{\xi }^2)(1/\rho _{{\hat{\eta }}_1}-1) +\gamma _2\sigma _{\zeta _1}^2/\rho _{{\hat{\eta }}_1} \end{array} \right) . \end{aligned}$$

Equations (10) and (11) relate the values of the regression coefficients under PAWC to those under SEM. When $\rho _{{\hat{\xi }}}=\rho _{{\hat{\eta }}_1}=1$, $b=\beta _1$ and $c=\gamma _2$. However, with ${\hat{\xi }}$ and ${\hat{\eta }}_1$ containing errors, the value of *b* is also affected by the values of $\gamma _1$ and $\gamma _2$ in addition to the value of $\beta _1$. In particular, *b* is not necessarily smaller than $\beta _1$ due to $\xi$ and $\eta _1$ being correlated when $\gamma _1\ne 0$.

It follows from Eqs. (10) and (11) that, under PAWC, the indirect effect of ${\hat{\xi }}$ on ${\hat{\eta }}_2$ via ${\hat{\eta }}_1$ is given by:

12 $$\begin{aligned} ab= \rho _{{\hat{\xi }}}\rho _{{\hat{\eta }}_1}\frac{\gamma _1[\gamma _1(\beta _1\gamma _1+\gamma _2)\sigma _{\xi } ^2(1-\rho _{{\hat{\xi }}})+\beta _1\sigma _{\zeta _1}^2]}{\sigma _{\zeta _1}^2+ \gamma _1^2\sigma _{\xi }^2(1-\rho _{{\hat{\xi }}}\rho _{{\hat{\eta }}_1})}. \end{aligned}$$

Equation (12) implies that $ab=\gamma _1\beta _1$ when $\rho _{{\hat{\xi }}}=\rho _{{\hat{\eta }}_1}=1$. But *ab* is not necessarily smaller than $\gamma _1\beta _1$ even when $\rho _{{\hat{\xi }}}$ and $\rho _{{\hat{\eta }}_1}$ are both less than 1.0.

For mediation analysis with weighted composites, the sample variances of the LS estimates $\hat{a}$ and $\hat{b}$ are obtained via the standard formula of regression analysis. Their population counterparts follow from the replacement of the sample variances-covariances by the population variances-covariances. Appendix B contains the details relating the variances of $\hat{a}$ and $\hat{b}$ to the base parameters in ${\varvec{\theta }}$. In particular, the variances of $\sqrt{N}\hat{a}$, $\sqrt{N}\hat{b}$ and $\sqrt{N}\hat{c}$ are, respectively, given by

13 $$\begin{aligned} \omega _{aw}^2=\frac{ \rho _{{\hat{\xi }}}\sigma _{\eta _1}^2}{(\rho _{{\hat{\eta }}_1}\sigma _{\xi }^2)}-\rho _{{\hat{\xi }}}^2\gamma _1^2, \;\; \omega _{bw}^2= \frac{\sigma _{e_2}^2}{\sigma _{\eta _1}^2/\rho _{{\hat{\eta }}_1}-\rho _{{\hat{\xi }}}\gamma _1^2\sigma _{\xi }^2}, \;\;\textrm{and}\;\; \omega _{cw}^2= \frac{\sigma _{e_2}^2\sigma _{\eta _1}^2}{\sigma _{\xi }^2\sigma _{\eta _1}^2/\rho _{{\hat{\xi }}} -\rho _{{\hat{\eta }}_1}\gamma _1^2\sigma _{\xi }^4}, \nonumber \\ \end{aligned}$$

where $\sigma _{e_2}^2$ is the variance of $e_2$ of the 2nd regression model in Eq. (7). The subscript *w* in Eq. (13) is to indicate that the variance is computed conditional on weights. As a function of the base parameters in ${\varvec{\theta }}$ the expression of $\sigma _{e_2}^2$ is rather complicated, and we leave it to Appendix B.

Conditional on weights, the population values of the SNRs of the LS estimates $\hat{a}$, $\hat{b}$ and $\hat{c}$ can be directly computed according to Eq. (1) via the formulas in Eqs. (10), (11) and (13) together with the results in Eqs. (B3), (B9) and (B10) of Appendix B, respectively. These will be compared against those of the NML estimates of $\gamma _1$, $\beta _1$ and $\gamma _2$ in the following section. Our main interest is the SNR for the estimate $\hat{a}\hat{b}$. It can be shown that $\hat{a}$ and $\hat{b}$ are independent when $\textbf{x}$, $\textbf{y}_1$ and $\textbf{y}_2$ are jointly normally distributed, while $\hat{b}$ and $\hat{c}$ are correlated in general. Using the delta-method (Ferguson, 1996), the variance of $\sqrt{N}\hat{a}\hat{b}$ is given by:

14 $$\begin{aligned} \omega _{abw}^2=a^2\omega _{bw}^2+b^2\omega _{aw}^2, \end{aligned}$$

which does not depend on *N* and can be computed via the formulas in Eqs. (10), (11) and (13). When evaluated at the output of path analysis, ${\hat{\omega }}_{abw}/\sqrt{N}$ is essentially the SE given by Sobel (1982). While the distribution of $\hat{a}\hat{b}$ is not symmetric, the SNR

15 $$\begin{aligned} \tau _{abw}=\frac{ab}{\omega _{abw}}=(1/\tau _{aw}^2+1/\tau _{bw}^2)^{-1/2} \end{aligned}$$

plays a pivotal role in the NHST of $ab=0$ regardless of the methods being used (e.g., bootstrap, Monte Carlo, asymptotics), where $\tau _{aw}=a/\omega _{aw}$ and $\tau _{bw}=b/\omega _{bw}$. We will refer to $\tau _{abw}$ in Eq. (15) as the population or asymptotic SNR under PAWC conditional on weights.

### Indirect Effect Under PAWC with Estimated Weights

Except for EWCs or studies where weights of composites can be assigned *a priori* or according to design, the optimal weights may need to be estimated from the observed data. Then sampling errors in the observed data will affect the properties of the weighted composites and the size of the resulting SNRs under PAWC. However, even for factor scores whose corresponding ${\hat{\textbf{w}_x}}$, ${\hat{\textbf{w}}}_{y_1}$ and ${\hat{\textbf{w}}}_{y_2}$ have analytical forms, quantifying the effect of sampling errors on the estimated weights and the resulting $\hat{a}\hat{b}$ is rather involved and is also beyond the immediate interest of the current article. Appendix C contains the development leading to a formula for computing the asymptotic variance of $\sqrt{N}\hat{a}\hat{b}$ when Bartlett-factor scores (BFSs) are used in PAWC, where weights are computed via the NML estimates of a confirmatory factor model. Let this variance be $\omega _{ab\hat{w}}^2$. Then, the corresponding SNR is given by

16 $$\begin{aligned} \tau _{ab\hat{w}}=\frac{ab}{\omega _{ab\hat{w}}}, \end{aligned}$$

where the $\hat{w}$ in the subscript indicates that sampling errors in ${\hat{\textbf{w}}}$ are accounted for in the formulation of $\tau _{ab\hat{w}}$. We will refer to $\tau _{ab\hat{w}}$ in Eq. (16) as the population or asymptotic SNR under PAWC with estimated weights.

## Numerical Comparison

In this section, we numerically compare the $\tau _{\gamma _1\beta _1}$ in Eq. (6), the $\tau _{abw}$ in Eq. (15), and the $\tau _{ab\hat{w}}$ in Eq. (16) as well as their empirical counterparts when the SDs in the three equations are obtained empirically. We will first describe the conditions under which the population values of the base parameters in ${\varvec{\theta }}$ are obtained, and then present the results of numerical comparison.

Equally weighted composites (EWCs) and factor scores (FSs) are most widely used in research (DiStefano et al., 2009). For items containing measurements errors, the properties of the EWCs are well understood in psychometrics (e.g., Allen & Yen, 1979; Widaman & Revelle, 2023). Properties and applications of FSs have also been thoroughly studied and documented (Hoshino and Bentler, 2013; Lawley and Maxwell, 1971; Schuster and Lubbe, 2020)). In particular, EWC and FS represent the two extremes of weighted composites, the former does not consider the psychometric properties of individual items at all whereas the later optimally assigns weights to items to achieve maximum reliability. As we have shown, the SNR for the indirect effect under PAWC depends on the reliability of the composites being used. Consequently, path analysis via FSs is expected to yield the greatest SNR on average, while path analysis with EWCs is expected to yield the least favorable SNRs among all PAWCs. Our study of PAWC is also via the two types of composites. Because Bartlett-factor scores (BFSs) and regression-factor scores are proportional to each other and are equivalent in conducting path analysis (Yuan and Deng, 2021), we will choose the BFSs in our study. There are other type of composites in addition to the EWCs and factor scores (see, e.g., Cho & Choi, 2020; Hwang et al., 2021; McDonald, 1996). While their psychometric properties are less known for measurements that contain errors, we expect that their reliabilities are somewhere between those of the EWCs and BFSs, and path analysis via these composites is expected to perform in between.

### Design of Conditions

While the number of indicators can be arbitrary in practice, most studies contain three or more indicators for each latent variable. Thus, we choose $p_x=p_{y_1}=p_{y_2}=3$. The results in Yuan and Fang (2023) suggest that regression analysis via weighted composites has more advantage over SEM as the number of indicators increases. Other numbers of indicators had also been used in our study and the results showed patterns as expected. That is, as the number of indicators increases, PAWC has even more advantage over SEM than in the results reported below. Appendix D contains supporting results with $p_x=p_{y_1}=p_{y_2}=10$, and additional discussions.

With 3 indicators for each latent variable, the model under SEM has $q=21$ base parameters. A random sample of size 21 is drawn from the uniform distribution on [0, 1]. By adding the value of 0.2 to each of these 21 numbers to avoid the case for a parameter value to be too close to zero, the resulting 21 values are used as the population values of the 21 base parameters. Using independent replications, $N_c=1000$ sets of population values of the base parameters are obtained. The $1000\times 21$ values of the base parameters together with the SAS code that generates the parameters are in supplementary material https://www3.nd.edu/~kyuan/mediation1000c/. Each set of 21 values represents a condition for the population ${\varvec{\theta }}$. Note that the population values of *a*, *b*, *c* as well as the variances of ${\hat{\xi }}$, ${\hat{\eta }}_1$, ${\hat{\eta }}_2$, $e_1$ and $e_2$ in Eq. (7) are determined by the 21 population values of the base parameters in ${\varvec{\theta }}$ according to the formulas developed in the previous section.

### Population SNR

We have two population $\tau$ for path analysis with the BFSs. One is conditional on weights held at the population values, and the other is by considering the errors in weights analytically. Thus, for each set of the population values of the 21 base parameters in ${\varvec{\theta }}$, four different $\tau$ are evaluated. They are (1) the SNR $\tau _{\gamma _1\beta _1}$ under SEM according to Eq. (6); (2) the SNR $\tau _{abw}$ under path analysis via Bartlett-factor scores with population weights (paBFS(*w*)) according to Eq. (15); (3) the SNR $\tau _{ab\hat{w}}$ under path analysis via Bartlett-factor scores with estimated weights (paBFS($\hat{w}$)) by Eq. (16); and (4) the SNR $\tau _{ab}$ under path analysis via equally weighted composites (paEWC) according to Eq. (15). Consequently, we have 1000 values of $\tau _{\gamma _1\beta _1}$, and 1000 values of $\tau _{ab}$ by paBFS(*w*), paBFS($\hat{w}$), paEWC, respectively. Note that the weights in paBFS(*w*) as well as the SDs for computing the population $\tau$s are all based on the true or population values of the 21 base parameters. Corresponding to the values of the SNR for the indirect effect, we also have 1000 values of the SNRs corresponding to ${\hat{\gamma }}_1$, ${\hat{\gamma }}_2$ and ${\hat{\beta }}_1$ as well as those corresponding to $\hat{a}$, $\hat{c}$ and $\hat{b}$, respectively. These are also based on the true/population values of the 21 base parameters. As we shall see, the ranges of the SNRs are rather wide, and we hope that they cover most of the conditions in empirical data analysis.

Table 1 contains the minimum value, maximum value, median, and the mean of the 1000 SNRs under each method. The parallel summary statistics for the SNRs corresponding to the estimates of $(\gamma _1, \gamma _2, \beta _1)$ and (*a*, *c*, *b*) are also reported in the table for additional information. For communication, the largest SNR value for each parameter among the four methods is put in bold while the smallest one is underlined. The SD and coefficient of variation (CV) of these SNRs across the 1000 conditions are included as well to understand the spread of the SNRs, although the differences among the values of the $\tau$ under each method are not due to sampling error. It follows from the first two columns of the numbers in Table 1 that the 1000 conditions cover a wide range of SNRs for each method. In particular, the range of $\tau _{\gamma _1\beta _1}$ is from 0.045 to 0.598; and those of $\tau _{ab}$ are 0.108 to 717, 0.109 to 0.865, and 0.104 to 0.701 corresponding to paBFS(*w*), paBFS($\hat{w}$), and paEWC, respectively.

**Table 1:** Population signal-to-noise ratios (SNR, $\tau$) by four methods across 1000 conditions: minimum, maximum, median, mean, standard deviation (SD) and coefficient of variation (CV).

Method	SNR	Min	Max	Median	Mean	SD	CV
SEM	$\tau _{\gamma _1}$	0.134	*0.989*	*0.470*	*0.472*	*0.179*	*0.379*
	$\tau _{\gamma _2}$	*0.032*	*0.812*	*0.269*	*0.297*	*0.155*	**0.522**
	$\tau _{\beta _1}$	*0.042*	*0.962*	*0.271*	*0.302*	*0.162*	**0.536**
	$\tau _{\gamma _1\beta _1}$	*0.045*	*0.598*	*0.237*	*0.251*	*0.105*	**0.418**
paBFS(*w*)	$\tau _a$	**0.137**	1.392	0.525	0.548	0.234	0.427
	$\tau _c$	**0.096**	1.199	**0.448**	**0.472**	0.189	0.400
	$\tau _b$	**0.110**	1.775	0.567	0.592	0.244	0.413
	$\tau _{ab}$	0.108	0.717	0.353	0.362	0.130	0.360
paBFS($\hat{w}$)	$\tau _a$	0.137	**2.096**	**0.527**	**0.590**	**0.307**	**0.520**
	$\tau _c$	0.094	**1.449**	0.436	0.464	**0.197**	0.425
	$\tau _b$	0.109	**2.643**	**0.568**	**0.616**	**0.289**	0.469
	$\tau _{ab}$	**0.109**	**0.865**	**0.366**	**0.382**	**0.150**	0.392
paEWC	$\tau _a$	*0.119*	1.266	0.482	0.502	0.213	0.424
	$\tau _c$	0.094	1.156	0.424	0.445	0.173	*0.388*
	$\tau _b$	0.106	1.650	0.543	0.567	0.230	*0.406*
	$\tau _{ab}$	0.104	0.701	0.336	0.342	0.123	0.361

SEM = structural equation modeling, paBFS (*w*) = path analysis by Bartlett-factor scores with population weights, paBFS ($\hat{w}$) = path analysis by Bartlett-factor scores with estimated weights, paEWC = path analysis with equally weighted composites.

The largest value among the four methods is in bold while the smallest is italicized.

According to Table 1, the SNRs by SEM have the smallest values in mean, median, and SD for the parameters $\gamma _1\beta _1 (ab)$, $\gamma _1 (a)$, $\gamma _2(c)$, and $\beta (b)$ across the 1000 conditions. The SNRs by SEM also have the smallest minimum and maximum values for $\gamma _1\beta _1 (ab)$, $\gamma _2(c)$, and $\beta (b)$ across the 1000 conditions. In contrast, the SNRs by paBFS($\hat{w}$) have the largest values in mean, median and SD for parameters $ab (\gamma _1\beta _1)$, $a (\gamma _1)$, and $b ( \beta )$, while the SNRs by paBFS(*w*) have the largest value in mean and median for the parameter $c(\gamma _2)$. The ratios of the median of $\tau _{ab}$ by paBFS(*w*), paBFS($\hat{w}$), and paEWC to that of $\tau _{\gamma _1\beta _1}$ are, respectively

17 $$\begin{aligned} \textrm{median}(\tau _{ab})/\textrm{median}(\tau _{\gamma _1\beta _1})=1.487, 1.544, 1.417; \end{aligned}$$

while the means of $\tau _{ab}$ by paBFS(*w*), paBFS($\hat{w}$), and paEWC to that of $\tau _{\gamma _1\beta _1}$ are respectively

18 $$\begin{aligned} \textrm{mean}(\tau _{ab})/\textrm{mean}(\tau _{\gamma _1\beta _1})=1.443, 1.523, 1.362. \end{aligned}$$

Thus, SEM is the least efficient method in estimating and testing the indirect effect. However, the SNRs by SEM for the indirect effect, $\gamma _2(c)$, and $\beta _1 (b)$ have the largest relative spread as reflected by the values of CV.

An interesting phenomenon is that $\tau _{ab\hat{w}}$ has greater mean and median than $\tau _{abw}$. This is because the estimated weights result in $\hat{a}\hat{b}$ that has a smaller SD in most of the conditions, due to the terms $\textbf{B}_{12}=\textbf{B}_{21}'$ in Eq. (C14) being negative. Similar phenomena were observed and discussed in Pierce (1982) and Yuan and Jennrich (2000). This is good news since we typically have to estimate the weights for the BFS in practice, and the property enhances our chance to detect the indirect effect.

**Table 2:** Pairwise comparison of the population signal-to-noise ratios (SNR, $\tau$) by four methods over 1000 conditions.

	$a(\gamma _1)$	$c(\gamma _2)$	$b(\beta _1)$	$ab(\gamma _1\beta _1)$
$\tau _{paBFS(w)}>\tau _{SEM}$	1000	1000	1000	894
$\tau _{paBFS(\hat{w})}>\tau _{SEM}$	914	997	1000	938
$\tau _{paEWC}>\tau _{SEM}$	676	995	999	786
$\tau _{paBFS(\hat{w})}>\tau _{paBFS(w)}$	721	366	681	867
$\tau _{paEWC}>\tau _{paBFS(w)}$	0	188	238	73
$\tau _{paEWC}>\tau _{paBFS(\hat{w})}$	87	333	226	73

SEM = structural equation modeling, paBFS (*w*) = path analysis by Bartlett-factor scores with population weights, paBFS ($\hat{w}$) = path analysis by Bartlett-factor scores with estimated weights, paEWC = path analysis with equally weighted composites.

The SNRs under each of the 1000 conditions are further compared across the different methods. Table 2 contains the frequency of the pairwise comparison on the values of the population SNRs. Across the 1000 conditions, the SNRs for $\gamma _1(a)$, $\gamma _2(c)$, and $\beta _1(b)$ by SEM are always smaller than those by paBFS(*w*). Across the 1000 conditions, SEM yields smaller SNRs for the indirect effect than paBFS(*w*), paBFS($\hat{w}$) and paEWC 894, 938 and 786 times, respectively. The method paBFS(*w*) yields smaller SNRs for the indirect effect than paBFS($\hat{w}$) 867 times. The results of pairwise comparison among the other methods in Table 2 are also consistent with those in Table 1.

The results in Tables 1 and 2 generalize the results in Yuan and Fang (2023), where, for a model with two latent variables, regression analysis via BFSs is shown to have mathematically greater SNR than SEM. While path analyses via weighted composites may not always yield greater SNR for the indirect effect than SEM, they outperform SEM for most of the conditions. In particular, even paEWC outperforms SEM 786 times out of the 1000 conditions, and the average SNR for the indirect effect under paEWC is 1.362 times that under SEM.

### Empirical SNR

For the validity of the asymptotic results presented in the previous subsection, we compare them against their empirical counterparts at $N=200$, which represents a relatively small sample size for proper use of the SEM methodology. The rationale for not considering an even smaller *N* is that SEM may meet non-convergences, which will interfere with the results of the study. In addition, since we already have 1000 conditions of population ${\varvec{\theta }}$, the condition of $N=200$ at each of the 1000 population ${\varvec{\theta }}$ not only allows us to see the effect of a finite *N* but also permits us a manageable scope of study for a thorough analysis.

For each of the $N_c=1000$ conditions described in Sect. 3.1, a sample of size $N=200$ is drawn from the corresponding normal population $N(\textbf{0},{\varvec{\Sigma }}_c)$, where ${\varvec{\Sigma }}_c$ is the covariance matrix corresponding to a given condition. For this sample, estimates ${\hat{\gamma }}_1$, ${\hat{\gamma }}_2$, ${\hat{\beta }}_1$ are obtained by the NML method of SEM, and so is the product term ${\hat{\gamma }}_1{\hat{\beta }}_1$. Bartlett-factor scores are computed via the NML estimates of factor loadings and error variances, and followed by LS estimation of the model in Eq. (7). Estimates of $\hat{a}$, $\hat{c}$ and $\hat{b}$ are consequently obtained and so is $\hat{a}\hat{b}$. Denote the estimate of a parameter by a particular method as ${\hat{\theta }}$. We replicated this process $N_r=1000$ times for each of the $N_c=1000$ conditions, resulting in independent values ${\hat{\theta }}_i$, $i=1$, 2, $\ldots$, $N_r$. The empirical SNRs are obtained as:

$$\begin{aligned} {\hat{\tau }}=\frac{{\bar{\theta }}}{\textrm{SD}_{\theta }}, \end{aligned}$$

where ${\bar{\theta }}=\sum _{i=1}^{N_r}{\hat{\theta }}_i/N_r$ and $\textrm{SD}_{\theta }=N^{1/2}\textrm{SE}_{\theta }$ with $\textrm{SE}_{\theta }=[\sum _{i=1}^{N_r}({\hat{\theta }}_i-{\bar{\theta }})^2/(N_r-1)]^{1/2}$. In addition to the empirical SNRs with estimated weights, we also computed the empirical SNRs with weights held at the population values under paBFS(*w*), and paEWC. Thus, we have total of four empirical SNRs at each of the $N_c$ conditions. They are, respectively, (1) SEM, (2) path analysis via BFS with population weights, (3) path analysis via BFS with estimated weights, and 4) path analysis with equally weighted composites.

**Table 3:** Empirical signal-to-noise ratios (SNR, ${\hat{\tau }}$) by four methods across 1000 conditions, and the ${\hat{\tau }}$ at each condition is evaluated via 1000 replications: minimum, maximum, median, mean, standard deviation (SD) and coefficient of variation (CV).

Method	SNR	Min	Max	Median	Mean	SD	CV
SEM	${\hat{\tau }}_{\gamma _1}$	0.131	*0.986*	*0.464*	*0.467*	*0.179*	*0.384*
	${\hat{\tau }}_{\gamma _2}$	*0.043*	*0.795*	*0.235*	*0.271*	*0.156*	**0.575**
	${\hat{\tau }}_{\beta _1}$	*0.049*	*0.914*	*0.237*	*0.275*	*0.159*	**0.579**
	${\hat{\tau }}_{\gamma _1\beta _1}$	*0.037*	*0.566*	*0.201*	*0.218*	*0.106*	**0.485**
paBFS(*w*)	${\hat{\tau }}_{a}$	0.133	1.401	**0.526**	0.544	0.232	0.427
	${\hat{\tau }}_c$	0.093	1.160	**0.445**	**0.468**	0.187	0.400
	${\hat{\tau }}_b$	0.107	1.771	0.562	0.587	0.243	0.415
	${\hat{\tau }}_{ab}$	0.105	0.717	0.348	0.358	0.130	0.364
paBFS($\hat{w}$)	${\hat{\tau _a}}$	*0.109*	**2.093**	0.521	**0.583**	**0.307**	**0.526**
	${\hat{\tau }}_c$	0.112	**1.448**	0.426	0.456	**0.196**	0.430
	${\hat{\tau }}_b$	**0.122**	**2.639**	**0.562**	**0.606**	**0.287**	0.473
	${\hat{\tau }}_{ab}$	0.118	**0.873**	**0.356**	**0.374**	**0.149**	0.397
paEWC	${\hat{\tau }}_a$	**0.133**	1.276	0.481	0.500	0.212	0.424
	${\hat{\tau }}_c$	**0.113**	1.132	0.422	0.441	0.171	*0.388*
	${\hat{\tau }}_b$	0.120	1.662	0.540	0.562	0.229	*0.407*
	${\hat{\tau }}_{ab}$	**0.118**	0.687	0.334	0.338	0.123	*0.363*

SEM = structural equation modeling, paBFS(*w*) = path analysis by Bartlett-factor scores with population weights, paBFS($\hat{w}$) = path analysis by Bartlett-factor scores with estimated weights, paEWC = path analysis with equally weighted composites.

The largest value among the four methods is in bold while the smallest is italicized.

Table 3 contains the summary statistics for the empirical SNRs over the $N_c=1000$ conditions. Parallel to Table 1, the largest value of each parameter among the four methods is boldfaced and the smallest one is underlined. According to Table 3, the empirical SNRs by SEM have the smallest mean and median across the 1000 conditions, while they have the largest CVs for three out of the four parameters. In Table 3, the ratios of median and mean of ${\hat{\tau }}_{ab}$ by paBFS$(\hat{w})$ over those of ${\hat{\tau }}_{\gamma _1\beta _1}$ are 1.769 and 1.716, respectively, greater than their asymptotic counterparts in Eqs. (17) and (18).

**Table 4:** Pairwise comparison of the empirical signal-to-noise ratios (SNR, ${\hat{\tau }}$) by four methods over 1000 conditions, and the ${\hat{\tau }}$ at each condition is evaluated via 1000 replications.

	$a(\gamma _1)$	$c(\gamma _2)$	$b(\beta _1)$	$ab(\gamma _1\beta _1)$
${\hat{\tau }}_{paBFS(w)}>{\hat{\tau }}_{SEM}$	989	1000	1000	945
${\hat{\tau }}_{paBFS(\hat{w})}>{\hat{\tau }}_{SEM}$	903	998	1000	975
${\hat{\tau }}_{paEWC}>{\hat{\tau }}_{SEM}$	729	994	999	861
${\hat{\tau }}_{paBFS(\hat{w})}>{\hat{\tau }}_{paBFS(w)}$	668	284	591	804
${\hat{\tau }}_{paEWC}>{\hat{\tau }}_{paBFS(w)}$	13	201	244	93
${\hat{\tau }}_{paEWC}>{\hat{\tau }}_{paBFS(\hat{w})}$	120	380	267	109

SEM = structural equation modeling, paBFS (*w*) = path analysis by Bartlett-factor scores with population weights, paBFS ($\hat{w}$) = path analysis by Bartlett-factor scores with estimated weights. paEWC = path analysis with equally weighted composites.

Table 4 contains the results of pairwise comparison of the empirical SNRs by the four methods. Out of the 1000 conditions, the values of ${\hat{\tau }}$ by SEM are smaller than those by paBFS(*w*) and paBFS$(\hat{w})$ 945 and 975 times, respectively. Even path analysis with equally weighted composites yields greater SNR (${\hat{\tau }}_{ab}$) than SEM 861 times out of the 1000 conditions. The three methods of PAWC also clearly outperform SEM for the other three parameters with respect to SNR.

The results in Tables 3 and 4 are consistent, and they showed that SEM is the least efficient/powerful method for the purpose of detecting the existence of an indirect effect in mediation analysis.

### Graphical Comparison

**Fig. 1:**
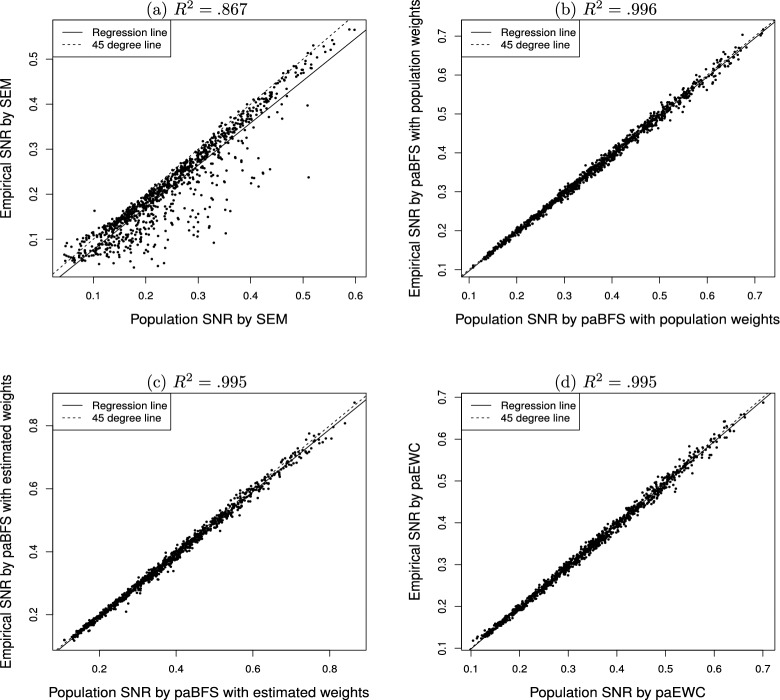
Empirical signal-to-noise ratio (SNR, $\hat{\tau }$) against population SNR ($\tau$) for estimating and testing the indirect effect in mediation analysis, over 1000 conditions.

For information about the overall distribution of the SNRs of the indirect effect over the $N_c=1000$ conditions, Fig. 1 contains the scatter plots of the empirical SNRs (${\hat{\tau }}$) at $N=200$ against their population/asymptotic counterparts ($\tau$). The dotted line in each plot is the $x=y$ line, and the solid line is the regression line of the values in the vertical axis against those in the horizontal axis. The *R*-square of the regression line is also included in the figure. Figure 1a indicates that there exist sizeable differences between the empirical ${\hat{\tau }}_{\gamma _1\beta _1}$ and their population counterpart $\tau _{\gamma _1\beta _1}$ for some conditions, with the majority satisfying ${\hat{\tau }}_{\gamma _1\beta _1}>\tau _{\gamma _1\beta _1}$. In contrast, for the three PAWC methods, the two versions of the SNR agree well at $N=200$. In particular, the *R*-squares for the three methods of PAWC are all above 0.99, although the weights under paBFS($\hat{w})$ were computed from a confirmatory factor model under the SEM method.

The larger differences between ${\hat{\tau }}_{\gamma _1\beta _1}$ and $\tau _{\gamma _1\beta _1}$ in Fig. 1a than those between ${\hat{\tau }}_{ab}$ and $\tau _{ab}$ in Figs. 1b–d reflect the sizes of sampling errors between the two classes of methods. Regression analysis yields parameter estimates that are unbiased, which is a finite-sample property. In contrast, SEM only yields parameter estimates that are consistent, which is an asymptotic property. The patterns in Fig. 1 suggest that we might need a much greater sample size for the SEM method to yield a ${\hat{\tau }}$ having the same accuracy as the PAWC counterpart.

**Fig. 2:**
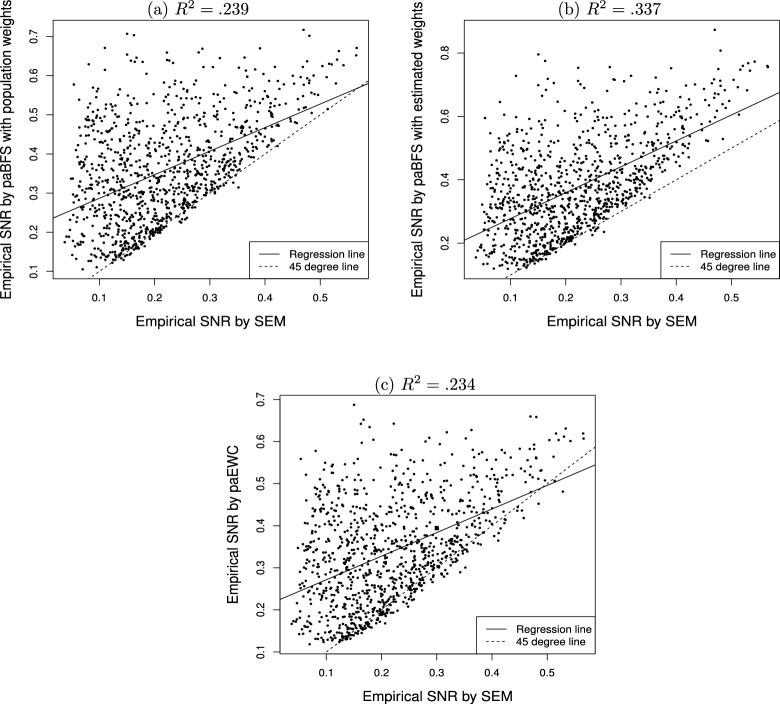
Empirical signal-to-noise ratio (SNR, $\hat{\tau }$) for estimating and testing the indirect effect in mediation analysis: Path analysis with weighted composites against SEM over 1000 conditions.

**Fig. 3:**
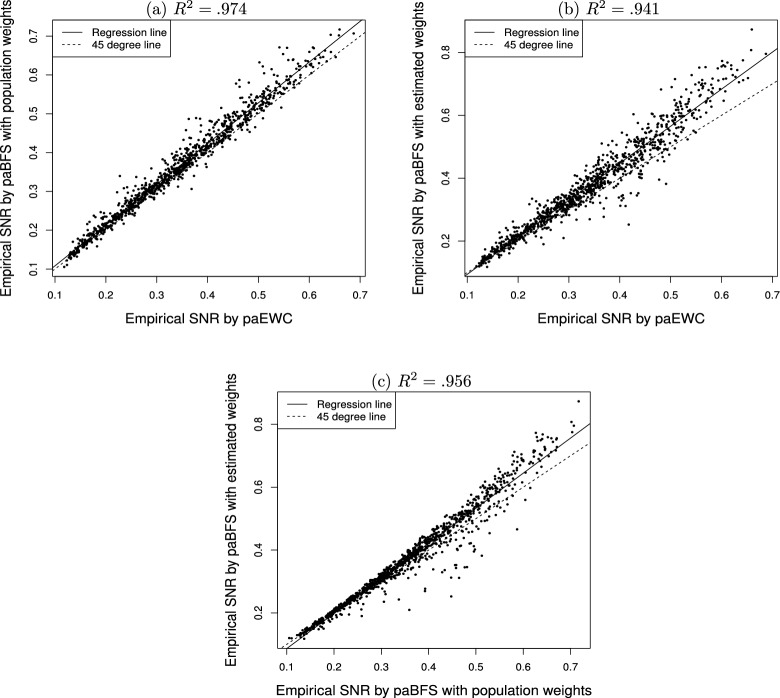
Empirical signal-to-noise ratio (SNR, $\hat{\tau }$) for estimating and testing the indirect effect in mediation analysis: Pairwise comparison of path analyses with weighted composites over 1000 conditions.

Figure 2 contains the scatter plots of ${\hat{\tau }}_{ab}$ against ${\hat{\tau }}_{\gamma _1\beta _1}$ over the 1000 conditions, where the dotted and solid lines serve the same functions as in Fig. 1. It is easy to see that most of the points in Fig. 2a–c are above the $x=y$ line, especially those in Fig. 2a and b. The values of the $R^2$ in Fig. 2 are rather small though, implying that many factors might contribute to the differences between ${\hat{\tau }}_{\gamma _1\beta _1}$ and ${\hat{\tau }}_{ab}$. In particular, ${\hat{\tau }}_{ab}$ can be much greater than ${\hat{\tau }}_{\gamma _1\beta _1}$ when the values of the later are small.

The values of ${\hat{\tau }}_{ab}$ among path analyses with differently weighted composites in Fig. 3 are rather close. The largest value of $R^2$ in Fig. 3 (0.974) is between paBFS(*w*) and paEWC, which might be because both their weights are held constant. Note that most of the points in each of the plots in Fig. 3 are above the $x=y$ line. But Fig. 3b and c contains points that are clearly below the $x=y$ line, indicating the existence of unfavorable conditions for paBFS($\hat{w}$) as compared against paBFS(*w*) and paEWC. We will further study the causes for the differences between ${\hat{\tau }}_{ab}$ and ${\hat{\tau }}_{\gamma _1\beta _1}$ in the next section.

## Causes of the SNR Differences

In this section, we examine parameters/factors that cause the difference $\tau _d=\tau _{ab}-\tau _{\gamma _1\beta _1}$ of the SNRs. We will use the empirical ${\hat{\tau }}_d={\hat{\tau }}_{ab}-{\hat{\tau }}_{\gamma _1\beta _1}$ to act as the representative of $\tau _d$ for the following reasons: (1) There exist sizeable differences between ${\hat{\tau }}_{\gamma _1\beta _1}$ and $\tau _{\gamma _1\beta _1}$ due to sampling errors. (2) By working with ${\hat{\tau }}_d$, we can directly study the causes of the observed differences, and the finding would be more informative in explaining the performances of different methods in practice.

Note that ${\hat{\tau }}_{ab}$ varies with the weighted composites used in conducting the mediation analysis while ${\hat{\tau }}_{\gamma _1\beta _1}$ is always computed following NML estimation of the SEM model. Without ambiguity, we distinguish the different ${\hat{\tau }}_d$ by the methods under which the composites are obtained, and our presentation and discussion are also labeled according to the corresponding PAWC methods. Because all the 21 base parameters may contribute to the values of the SNRs of $\hat{a}$, $\hat{b}$ as well as those of the consequent $\hat{a}\hat{b}$, we will only identify those that have most salient effect on ${\hat{\tau }}_d$. For such a purpose, we also include candidates that are known to affect the values of *a*, *b* and the SDs of their estimates. They are the reliability coefficients of ${\hat{\xi }}$, ${\hat{\eta }}_1$ and ${\hat{\eta }}_2$, the reliability coefficients of the nine individual indicators, and the four SNRs of the involved parameter estimates ${\hat{\gamma }}_1$, ${\hat{\beta }}_1$, $\hat{a}$ and $\hat{b}$. So there are a total of 37 (=21+3+9+4) potential predictors of ${\hat{\tau }}_d$. We use the population values of these potential predictors for more valid results and will refer them as covariates. Note that the 21 base parameters in ${\varvec{\theta }}$ and the 9 individual reliability coefficients do not change with the formulations of the composites. But the SNRs of $\hat{a}$ and $\hat{b}$ change with the weighting method and so do the reliability coefficients of ${\hat{\xi }}$, ${\hat{\eta }}_1$ and ${\hat{\eta }}_2$. We will use $\tau _{\gamma _1}$, $\tau _{\beta _1}$, $\tau _a$, and $\tau _b$ to denote the SNRs corresponding to ${\hat{\gamma }}_1$, ${\hat{\beta }}_1$, $\hat{a}$, and $\hat{b}$, respectively.

**Table 5:** Covariates that have squared correlations with the empirical SNR difference (${\hat{\tau }}_d={\hat{\tau }}_{ab}-{\hat{\tau }}_{\gamma _1\beta _1}$) greater than 0.05 across 1000 conditions, the listed method corresponds to ${\hat{\tau }}_{ab}$, while ${\hat{\tau }}_{\gamma _1\beta _1}$ always corresponds to SEM.

Covariate	paBFS (*w*)	paBFS ($\hat{w}$)	paEWC
$\gamma _1$	0.732	0.776	0.726
$\tau _a$	0.687	0.773	0.666
$\tau _{\gamma _1}$	0.678	0.782	0.671
$\rho _{y_1}$	0.302	0.340	0.254
$\rho _{{\hat{\eta }}_1}$	0.244	0.293	(0.220)
$\rho _{{\hat{\eta }}_2}$	0.230	0.284	0.228
$\rho _{y_4}$	(0.220)	0.268	(0.187)
$\tau _b$	(0.207)	0.268	(0.187)
$\rho _{{\hat{\xi }}}$	−0.253	(−0.094)	−0.250
$\sigma _{\zeta _1}^2$	−0.437	−0.389	−0.449
$\tau _{\beta _1}$	−0.437	−0.298	−0.486

The squared values of the numbers in the parentheses are less than 0.05. paBFS (*w*) = path analysis by Bartlett-factor scores with population weights, paBFS ($\hat{w}$) = path analysis by Bartlett-factor scores with estimated weights, paEWC = path analysis with equally weighted composites. The listed covariates are identified from a total of 37 candidates of population parameters. The SNRs $\tau _a$ and $\tau _b$ as well as the reliabilities $\rho _{{\hat{\xi }}}$, $\rho _{{\hat{\eta }}_1}$ and $\rho _{{\hat{\eta }}_2}$ are evaluated according to the weights under each method.

The correlation between ${\hat{\tau }}_d$ and each of the 37 covariates across the 1000 conditions was computed first, and covariates that have squared correlations ($r^2$) greater than 0.05 are reported^2^ in Table 5. There are 9, 10, and 8 covariates with $r^2>.05$ for methods paBFS(*w*), paBFS($\hat{w}$) and paEWC, respectively. For a balanced table and/or fine information, the correlations of the shared covariates with $r^2\le .05$ are put in parentheses. According to the table, parameters $\gamma _1$, $\tau _{\gamma _1}$, and $\tau _a$ correlate most with ${\hat{\tau }}_d$ for all the three methods, although the orders of the size of their correlations are different. Such a pattern is expected since $\tau _d=0$ when $\gamma _1$, $\tau _{\gamma _1}$ or $\tau _a$ equal 0. However, $\tau _{\beta _1}$ is negatively correlated with ${\hat{\tau }}_d$ whereas $\tau _b$ is positively correlated with ${\hat{\tau }}_d$. Such a phenomenon might be due to the effect of the error term $\zeta _1$ whose variance is negatively correlated with ${\hat{\tau }}_d$. Note that $y_1$ is the anchor^3^ for $\eta _1$ under SEM, and the reliability of the anchor ($\rho _{y_1}$) has been shown to be inversely proportional to the sizes of the SEs of ${\hat{\gamma }}_1$ and ${\hat{\beta }}_1$ (Yuan and Zhang, 2023). Table 5 indicates that a greater $\rho _{y_1}$ also enhances the advantage of PAWC.

The reason for ${\hat{\tau }}_d$ to be negatively correlated with $\sigma _{\zeta _1}^2$ is because ${\hat{\tau }}_{ab}$ decreases faster than ${\hat{\tau }}_{\gamma _1\beta _1}$ as $\sigma _{\zeta _1}^2$ increases, although ${\hat{\tau }}_{\gamma _1\beta _1}$ also decreases at the same time. It can be shown analytically that both the variances of ${\hat{\gamma }}_1$ and $\hat{a}$ go to infinity as $\sigma _{\zeta _1}^2$ increases while holding other parameters constant. It is interesting that ${\hat{\tau _d}}$ correlates positively with $\rho _{{\hat{\eta }}_1}$ and $\rho _{{\hat{\eta }}_2}$ but negatively with $\rho _{{\hat{\xi }}}$. Results by separate calculation indicate that $\rho _{{\hat{\xi }}}$, $\rho _{{\hat{\eta }}_1}$ and $\rho _{{\hat{\eta }}_2}$ are positively correlated with both ${\hat{\tau }}_{\gamma _1\beta _1}$ and ${\hat{\tau }}_{ab}$. However, the correlations of $\rho _{{\hat{\eta }}_1}$ and $\rho _{{\hat{\eta }}_2}$ with ${\hat{\tau }}_{ab}$ are greater than with ${\hat{\tau }}_{\gamma _1\beta _1}$ while it is the opposite for $\rho _{{\hat{\xi }}}$. Similarly, $\tau _{\beta _1}$, $\gamma _1$, $\tau _{\gamma _1}$, $\tau _a$, $\tau _b$, $\rho _{y_1}$ and $\rho _{y_4}$ are also positively correlated with both ${\hat{\tau }}_{\gamma _1\beta _1}$ and ${\hat{\tau }}_{ab}$, and the correlation of $\tau _{\beta _1}$ with ${\hat{\tau }}_{\gamma _1\beta _1}$ is greater than with ${\hat{\tau }}_{ab}$ while it is the opposite for the other covariates.

**Table 6:** Results for the five best subsets of predictors of the empirical SNR differences ${{\hat{\tau }_d}}={\hat{\tau }}_{ab}-{\hat{\tau }}_{\gamma _1\beta _1}$, and the listed method corresponds to ${\hat{\tau }}_{ab}$ while ${\hat{\tau }}_{\gamma _1\beta _1}$ is always obtained under SEM.

	paBFS (*w*)			paBFS($\hat{w}$)			paEWC		
#pre	$R^2$	Pred	Coef	$R^2$	Pred	Coef	$R^2$	Pred	Coef
1	0.536	1	−0.074	0.611	1	−0.097	0.528	1	−0.085
		$\gamma _1$	0.308		$\tau _{\gamma _1}$	0.536		$\gamma _1$	0.295
2	0.720	1	0.052	0.728	1	0.120	0.722	1	0.040
		$\gamma _1$	0.306		$\rho _{{\hat{\xi }}}$	−0.356		$\gamma _1$	0.293
		$\sigma _{\zeta _1}^2$	−0.179		$\tau _{\gamma _1}$	0.609		$\sigma _{\zeta _1}^2$	−0.178
3	0.786	1	0.229	0.761	1	0.179	0.786	1	0.190
		$\rho _{{\hat{\xi }}}$	−0.254		$\rho _{{\hat{\xi }}}$	−0.339		$\rho _{{\hat{\xi }}}$	−0.233
		$\gamma _1$	0.308		$\tau _{\gamma _1}$	0.575		$\gamma _1$	0.294
		$\sigma _{\zeta _1}^2$	−0.178		$\sigma _{\zeta _1}^2$	−0.079		$\sigma _{\zeta _1}^2$	−0.175
4	0.804	1	0.193	0.780	1	0.143	0.875	1	0.002
		$\rho _{{\hat{\xi }}}$	−0.261		$\rho _{{\hat{\xi }}}$	−0.346		$\tau _{\gamma _1}$	0.262
		$\gamma _1$	0.309		$\tau _{\gamma _1}$	0.576		$\tau _{\beta _1}$	−0.646
		$\gamma _2$	0.055		$\gamma _2$	0.057		$\tau _b$	0.371
		$\sigma _{\zeta _1}^2$	−0.177		$\sigma _{\zeta _1}^2$	−0.079		$\sigma _{\zeta _1}^2$	−0.030
5	0.810	1	0.163	0.794	1	0.101	0.878	1	0.018
		$\rho _{{\hat{\xi }}}$	−0.257		$\rho _{{\hat{\xi }}}$	−0.230		$\tau _{\gamma _1}$	0.159
		$\gamma _1$	0.283		$\tau _{\gamma _1}$	0.295		$\tau _{\beta _1}$	−0.582
		$\gamma _2$	0.054		$\gamma _1$	0.169		$\tau _b$	0.330
		$\sigma _{\zeta _1}^2$	−0.196		$\gamma _2$	0.061		$\gamma _1$	0.077
		$\rho _{y_1}$	0.093		$\sigma _{\zeta _1}^2$	−0.118		$\sigma _{\zeta _1}^2$	−0.055

paBFS(*w*)=path analysis by Bartlett-factor scores with population weights, paBFS($\hat{w}$)=path analysis by Bartlett-factor scores with estimated weights, paEWC=path analysis with equally weighted composites.

Note that $\gamma _1$, $\tau _{\gamma _1}$ and $\tau _{a}$ are closely related and so are $\tau _{\beta _1}$ and $\tau _{b}$. Similarly, $\rho _{y_1}$ and $\rho _{{\hat{\eta }}_1}$ are strongly correlated and so are $\rho _{y_4}$ and $\rho _{{\hat{\eta }}_2}$. The covariates identified in Table 5 do not contribute to the values of ${\hat{\tau }}_d={\hat{\tau }}_{ab}-{\hat{\tau }}_{\gamma _1\beta _1}$ independently. We next identify the covariates that jointly affect the values of ${\hat{\tau }}_d$ most. We use best-subset regression for such a purpose, and predictors are selected from the 37 covariates (21 base parameters, 4 component SNRs, and 12 reliability coefficients). Table 6 contains the results of the top five best subsets in predicting ${\hat{\tau }}_d$ corresponding to the methods paBFS(*w*), paBFS($\hat{w}$) and paEWC. For each subset, Table 6 also includes the list of the selected predictors, the corresponding regression coefficients, and the value of the $R^2$ of the resulting regression model. As expected, covariate $\tau _{\gamma _1}$ is first selected for paBFS($\hat{w}$) while $\gamma _1$ is first selected for the other two methods. The list of most effective predictors changes as more covariates enter the model. For all the models, $\gamma _1$, $\tau _{\gamma _1}$, and $\tau _b$ positively predict ${\hat{\tau }}_d$, whereas $\rho _{{\hat{\xi }}}$, $\sigma _{\zeta _1}^2$, and $\tau _{\beta _1}$ negatively predict ${\hat{\tau }}_d$. These are consistent with the bivariate relationships reported in Table 5. A new covariate in Table 6 is $\gamma _2$, which positively predicts the value of ${\hat{\tau }}_d$ weakly when ${\hat{\tau }}_{ab}$ is estimated by paBFS(*w*) and paBFS($\hat{w}$). With 5 predictors, the $R^2$ for the three methods are respectively 0.810, 0.794 and 0.878, implying that major variance of ${\hat{\tau }}_d$ has been accounted for by the identified predictors.

**Fig. 4:**
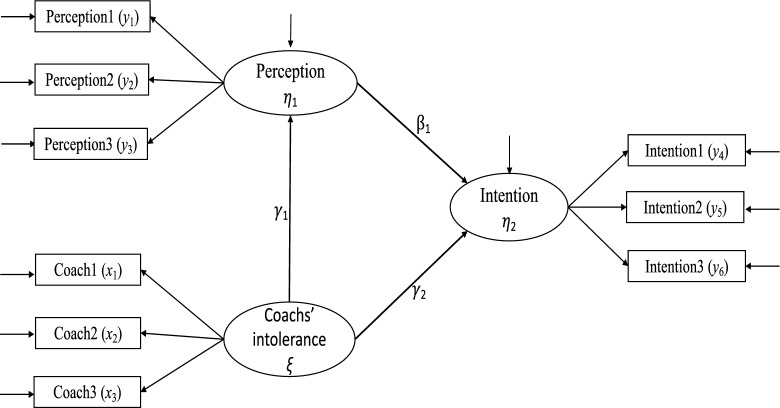
Coachs’ intolerance of steroids affect players’ intention via their perception.

The covariates being selected most frequently in Table 6 are $\sigma _{\zeta _1}^2$ (11 times), followed by $\gamma _1$ (10 times), $\rho _{{\hat{\xi }}_1}$ (8 times), $\tau _{\gamma _1}$ (7 times), and $\gamma _2$ (4 times). Except for the new covariate $\gamma _2$, all the coefficients in Table 6 also have the same signs as those for the bivariate correlations in Table 5.

According to the results in Table 6, path analyses with weighted composites tend to outperform SEM with respect to SNR for the indirect effect when values of $\gamma _1$, $\tau _{\gamma _1}$ or $\tau _a$ are large, while SEM might perform reasonably well when the values of $\sigma _{\zeta _1}^2$ and $\rho _{{\hat{\xi }}}$ are large. However, it is rare for SEM to yield a greater SNR than paBFS(*w*) or paBFS($\hat{w}$), although it is more likely for SEM to be superior to paEWC.

## A Real-Data Example

MacKinnon (2008) contains an example that studies the mediation effect of the extent to which coaches’ intolerance of steroids affects players’ intentions to use steroids via their perceptions about the lack of severity of steroid use. With nine variables, the data were from $N=547$ high school football players who were measured before the football season, immediately after the season, and several months after the end of the season. Table 7.3 of MacKinnon (2008) contains the EQS (Bentler, 2006) code for estimating the mediation model with three constructs, *coachs’ intolerance*, *perceived lack of severity of steroid use*, and *intention to use steroids*. Each construct is measured by three indicators. According to MacKinnon, *Coachs’ intolerance* was measured at time 1 and by questionnaire items: coach1–I have talked with at least one of my coaches about different ways to get stronger instead of using steroids, coach2–On my team there are rules against using steroids, and coach3–If I were caught using steroids, I would be in trouble with my coaches. The construct *perceived lack of severity* of steroid use was measured at time 2 and by questionnaire items: perception1–The bad effects of anabolic steroids go away as soon as you stop using them, perception2–Only a few people who use anabolic steroids ever have any harmful or unpleasant side effects, and perception3–Anabolic steroids are not dangerous if you use them only a few months each year. The construct *intention to use* steroids was measured at time 3 by items: intent1–I intend to try or use anabolic steroids, intent2–I would be willing to use anabolic steroids to know how it feels, and intent3–I am curious to try anabolic steroids. The path diagram representing the latent-variable model is given in Fig. 4, which is a graphical representation of the EQS code in Table 7.3 of MacKinnon (2008).

**Table 7:** The data (sample correlation matrix and standard deviations) and model are from Table 7.3 of MacKinnon (2008, $p=9$, $N=547$).

(a) Parameters estimates of the confirmatory factor model, their SEs and *z*-statistics ($T_{ml}=29.111$, $df=24$, CFI = 0.997, RMSEA = 0.020).												
$\theta$	est	se	*z*	$\theta$	est	se	*z*					
$\lambda _{x_1\xi }$	0.689	0.091	7.569	$\psi _{x11}$	3.258	0.206	15.814					
$\lambda _{x_2\xi }$	1.202	0.083	14.449	$\psi _{x22}$	0.907	0.161	5.650					
$\lambda _{x_3\xi }$	1.021	0.070	14.506	$\psi _{x33}$	0.633	0.115	5.491					
$\lambda _{y_1\eta _1}$	0.987	0.059	16.781	$\psi _{y11}$	1.100	0.081	13.566					
$\lambda _{y_2\eta _1}$	1.160	0.059	19.549	$\psi _{y22}$	0.860	0.081	10.641					
$\lambda _{y_3\eta _1}$	1.252	0.057	22.081	$\psi _{y33}$	0.529	0.078	6.778					
$\lambda _{y_4\eta _2}$	0.895	0.045	19.856	$\psi _{y44}$	0.632	0.044	14.469					
$\lambda _{y_5\eta _2}$	1.315	0.048	27.541	$\psi _{y55}$	0.222	0.047	4.760					
$\lambda _{y_6\eta _2}$	1.341	0.055	24.579	$\psi _{y66}$	0.561	0.058	9.728					
$\phi _{\eta _1\xi }$	-0.289	0.050	-5.831									
$\phi _{\eta _2\xi }$	-0.084	0.050	-1.666									
$\phi _{\eta _2\eta _1}$	0.293	0.045	6.539									

(b) Estimates of the coefficients of the structural model, their SEs and *z*-statistics by four different methods.												
	SEM			paBFS(*w*)			paBFS($\hat{w}$)			paEWC		
$\theta$	est	se	*z*	est	se	*z*	est	se	*z*	est	se	*z*
$\gamma _1 (a)$	−0.286	0.053	−5.371	−0.220	0.039	−5.608	−0.223	0.039	−5.739	−0.216	0.036	−5.989
$\gamma _2 (c)$	0.001	0.047	0.020	−0.009	0.035	−0.257	−0.010	0.039	−0.258	−0.028	0.032	−0.866
$\beta _1 (b)$	0.266	0.048	5.534	0.222	0.037	6.025	0.245	0.040	6.205	0.209	0.037	5.639
$\gamma _1\beta _1 (ab)$	−0.076	0.019	−3.980	−0.049	0.012	−4.105	−0.055	0.013	−4.160	−0.045	0.011	−4.106

SEM = structural equation modeling, paBFS (*w*) = path analysis by Bartlett-factor scores with population weights, paBFS ($\hat{w}$) = path analysis by Bartlett-factor scores with estimated weights, paEWC=path analysis with equally weighted composites.

Following MacKinnon (2008), we will treat the sample covariance matrix as from a normally distributed population. We first fit a confirmatory factor model (CFM) to the nine variables, and the CFM is statistically equivalent to the mediation model in Fig. 4. The likelihood ratio statistic and fit indices for the CFM are $T_{ml}=29.111$ ($df=24$), CFI=.997 (Bentler, 1990) and RMSEA = 0.020 (Steiger and Lind, 1980), indicating that the model fits the data very well. The top part of Table 7 contains the estimates of the parameters of the model, where each factor variance is fixed at 1.0. With the same notation for latent variables as in Eq. (2), all the estimated factor loadings ($\lambda$) and error variances ($\psi$) are statistically significant at 0.05 level according to $z\sim N(0,1)$, whereas the estimate of the correlation between $\eta _2$ and $\xi$ is not significant ($z_{\phi _{\eta _2\xi }}=-1.666$).

The estimates of the factor loadings and error variances are further used to compute Bartlett-factor scores, and PAWCs are subsequently conducted. The *z*-statistic for each path coefficient in the regression models (see Eq. 7) are obtained, so are those for the indirect effect $\hat{a}\hat{b}$, with SEs being computed using the delta method. Based on the Monte Carlo results in the previous section with $N=200$, the asymptotic SEs should provide good approximation to the true SEs at $N = 547$. We will not separately reporting the empirical SNRs for this example, because they can be easily obtained by dividing the absolute value of the *z*-statistics by $\sqrt{N}$.

The lower part of Table 7 contains the results of mediation analysis by the four methods examined previously. All the *z*-statistics for the indirect effect $\gamma _1\beta _1(ab)$ are significant, and the one by SEM is the smallest. The largest *z*-statistic or SNR for the indirect effect is given by paBFS($\hat{w}$). The *z*-statistics for parameters $\gamma _1(a)$ and $\beta _1(b)$ by SEM in Table 7 are also the smallest. It is interesting that $\hat{c}$ and ${\hat{\gamma }}_2$ have different signs, while neither of them is statistically significant. Such a phenomenon might deserve a focused study.

The results in Table 7 follow the same pattern as presented in Tables 1 to 4. That is, the SEM method does not show an advantage over PAWC for mediation analysis in terms of SNR or the *z*-statistic.

## Conclusion and Discussion

Based on the fact that values of the parameters in a latent-variable model are determined by scales of the latent variables that are subjectively assigned, recent literature has pointed out that bias in parameter estimates by PAWC is artificial rather than substantive. Consequently, for the purpose of confirming the existence of an indirect effect in conducting mediation analysis, methods should be evaluated according to their corresponding SNRs. To advance knowledge in this direction, we showed that PAWC tends to yield greater SNR for the indirect effect than the SEM methodology. In particular, paBFS ($\hat{w}$) most likely yield greater SNRs for the indirect effect than SEM. We also examined parameters/factors that cause SEM to have smaller SNRs, and results indicate that the advantage of PAWC becomes more obvious when there is a strong relationship between the latent predictor and the mediator, whereas the size of the prediction error in the mediator adversely affects the performance of the PAWC methodology.

SEM and PAWC are two widely used classes of methods in social and behavioral sciences. The strength of SEM is in yielding test statistics that convey the goodness of the overall model structure as well as consistent parameter estimates governing the structural relationship of the latent variables and indicators, assuming the model is correctly specified. The strength of PAWC is in yielding parameter estimates with larger SNRs and in prediction of individuals. Even if prediction or endorsing a mediation relationship is of primary interest, SEM still offers valuable information regarding the structural validity and reliability of individual indicators, as well as their dimensionality. These are key features of a measurement scale. For researchers who are interested in mediation analysis and also in the overall structure of the indicators, we recommend applying path analysis with the Bartlett-factor scores following the analysis under SEM.

Note that Bartlett-factor scores and regression-factor scores enjoy a property of attaining the maximum reliability. Because the reliabilities of composites are positively related to the SNR of the indirect effect and the coefficient of determination ($R^2$) for the regression models in Eq. (7), we suspect that other effect size measures in mediation analysis (e.g., Fairchild et al., 2009; Liu et al., 2024; MacKinnon, 2008; Lachowicz et al., 2018; Preacher & Kelley, 2011) also benefit from the use of the Bartlett- or regression-factor scores.

We only studied SNRs under the normality assumption for the observed variables. With nonnormally distributed data, SEs of parameter estimates under SEM can be strongly affected by the distribution of the data, especially heavy tails. SEs of the regression coefficients are also affected by nonnormality of the sample, but the effect is lessened by averaging over the indicators. We expect that PAWC will have even more advantage over SEM when data are of heavy-tails, while more research is needed in this direction.

For variables without predefined metrics, we noted that the values of parameters or their estimates between SEM and PAWC are not comparable, due to arbitrary rescaling by individual researchers. A commonly used technique is to standardize all the variables with the hope that the resulting parameter estimates become comparable. Standardizations might facilitate result comparison across different studies with the same dataset or different samples targeted for the same population within SEM or within the PAWC framework. However, the technique does not facilitate result comparison between SEM and PAWC, because standardizing a manifest variable under PAWC does not necessarily correspond to a proper standardization of the latent variable whenever measurement error exists, as implied by $x=\xi +e_x$. In addition, whenever variables have predefined metrics, standardizations often render results that are hard to interpret, as in a model for the relationship between weight and heights. More discussion on the issues are given in Yuan and Zhang (2023).

We did not study the effect of model misspecification on $\tau _{\gamma _1\beta _1}$ and $\tau _{ab}$. This is because SEM has the option of specifying cross-loadings and error-covariances but PAWC does not. The inclusion of cross-loadings or error-covariances can increase or decrease the value of the path coefficients among the latent variables (Yuan et al. 2003), whereas PAWC will have to reflect the additional association via the path coefficients. Thus, it is not a fair comparison for the two classes of methods when error covariances or cross-loadings are included in the latent-variable model. But it would be informative to study the effect of model misspecification on the SNRs when both classes of models ignore correlated errors or cross-loadings.

In this article, we only studied composites whose weights are explicit functions of the base parameters. There are other types of composites whose weights are implicit functions of the base parameters (see, e.g., Cho & Choi, 2020; Hwang et al., 2020; McDonald, 1996). We suspect that path analysis with these composites also yields greater average SNRs than SEM in estimating and testing the indirect effect, while their performances may vary, depending on the reliabilities of the particular composites. Additional studies are needed for more refined comparisons.

## Reliabilities of Weighted Composites

   This appendix contains the formulas for computing the variances of $\eta _1$ and $\eta _2$, and the reliability coefficients of weighted composites ${\hat{\xi }}$, ${\hat{\eta }}_1$ and ${\hat{\eta }}_2$. These are used to evaluate the SNR $\tau _{ab}$ under PAWC. Throughout the three appendices, we use $\sigma ^2$ and $\omega ^2$ for variances and $\sigma$ and $\omega$ for covariances, with the subscripts indicating the involved variables or the corresponding parameters being estimated.

   It follows from Eq. (3) and the independence of $\zeta _1$ and $\zeta _2$ with the corresponding predictors in the same equation that the variances of $\eta _1$ and $\eta _2$ are given by

$$\begin{aligned} \sigma _{\eta _1}^2=\gamma _1^2\sigma _{\xi }^2+\sigma _{\zeta _1}^2, \end{aligned}$$

and

$$\begin{aligned} \begin{array}{ll} \sigma _{\eta _2}^2&{}=\beta _1^2\sigma _{\eta _1}^2+\gamma _2^2\sigma _{\xi }^2+2\beta _1\gamma _2 \textrm{Cov}(\eta _1,\xi )+\sigma _{\zeta _2}^2\\ &{}=\beta _1^2(\gamma _1^2\sigma _{\xi }^2+\sigma _{\zeta _1}^2)+\gamma _2^2\sigma _{\xi }^2 +2\beta _1\gamma _1\gamma _2\sigma _{\xi }^2+\sigma _{\zeta _2}^2\\ &{}= (\beta _1\gamma _1+\gamma _2)^2\sigma _{\xi }^2 +\beta _1^2\sigma _{\zeta _1}^2+\sigma _{\zeta _2}^2. \end{array} \end{aligned}$$

   According to Eq. (2) and the formulations of weighted composites introduced in Sect. 2.3, we have

$$\begin{aligned}{} & {} {\hat{\xi }}=\frac{\textbf{w}_x'\textbf{x}}{\textbf{w}_x'{\varvec{\lambda }}_x}=\xi +\frac{\textbf{w}_x'\textbf{e}_x}{\textbf{w}_x'{\varvec{\lambda }}_x}, \\{} & {} \quad {\hat{\eta }}_1=\frac{\textbf{w}_{y_1}'\textbf{y}_1}{\textbf{w}_{y_1}'{\varvec{\lambda }}_{y_1}}=\eta _1+\frac{\textbf{w}_{y_1} '\textbf{e}_{y_1}}{\textbf{w}_{y_1}'{\varvec{\lambda }}_{y_1}}, \\{} & {} \quad {\hat{\eta }}_2=\frac{\textbf{w}_{y_2}'\textbf{y}_2}{\textbf{w}_{y_2}'{\varvec{\lambda }}_{y_2}} =\eta _2+\frac{\textbf{w}_{y_2}'\textbf{e}_{y_2}}{\textbf{w}_{y_2}'{\varvec{\lambda }}_{y_2}}. \end{aligned}$$

Standard covariance algebra shows that the reliabilities of ${\hat{\xi }}$, ${\hat{\eta }}_1$ and ${\hat{\eta }}_2$ are, respectively, given by

$$\begin{aligned}{} & {} \rho _{{\hat{\xi }}}=\frac{\sigma _{\xi }^2}{\sigma _{\xi }^2+\textbf{w}_x'{\varvec{\Psi }}_x\textbf{w}_x/(\textbf{w}_x'{\varvec{\lambda }}_x)^2} =\frac{\sigma _{\xi }^2(\textbf{w}_x'{\varvec{\lambda }}_x)^2}{\sigma _{\xi }^2(\textbf{w}_x'{\varvec{\lambda }}_x)^2+\textbf{w}_x'{\varvec{\Psi }}_x\textbf{w}_x}, \\{} & {} \quad \rho _{{\hat{\eta }}_1}=\frac{\sigma _{\eta _1}^2}{\sigma _{\eta _1}^2+\textbf{w}_{y_1}'{\varvec{\Psi }}_{y_1}\textbf{w}_{y_1}/(\textbf{w}_{y_1} '{\varvec{\lambda }}_{y_1})^2}= \frac{\sigma _{\eta _1}^2(\textbf{w}_{y_1}'{\varvec{\lambda }}_{y_1})^2}{\sigma _{\eta _1}^2(\textbf{w}_{y_1}'{\varvec{\lambda }}_{y_1})^2+\textbf{w}_{y_1} '{\varvec{\Psi }}_{y_1}\textbf{w}_{y_1}}, \end{aligned}$$

and

$$\begin{aligned} \rho _{{\hat{\eta }}_2}=\frac{\sigma _{\eta _2}^2}{\sigma _{\eta _2}^2+\textbf{w}_{y_2}' {\varvec{\Psi }}_{y_2}\textbf{w}_{y_2}/(\textbf{w}_{y_2}'{\varvec{\lambda }}_{y_2})^2}= \frac{\sigma _{\eta _2}^2(\textbf{w}_{y_2}'{\varvec{\lambda }}_{y_2})^2}{\sigma _{\eta _2}^2(\textbf{w}_{y_2} '{\varvec{\lambda }}_{y_2})^2+\textbf{w}_{y_2}'{\varvec{\Psi }}_{y_2}\textbf{w}_{y_2}}. \end{aligned}$$

Note that we fixed the variance of $\xi$ and the loadings of the first indicators in $\textbf{y}_1$ and $\textbf{y}_2$ at 1.0 in the development of the article, and the value of 1.0 and the chosen indicators do not affect the values of the three reliability coefficients.

## Asymptotic Variances of $\hat{a}$, $\hat{b}$, $\hat{c}$ and $\hat{a}\hat{b}$ by PAWC Conditional on Weights

   In this appendix, we derive the formulas for the asymptotic variances of $\hat{a}$, $\hat{b}$, $\hat{c}$ and $\hat{a}\hat{b}$ by relating them to the base parameters in ${\varvec{\theta }}$ under Eqs. (2) and (3). Formulas for evaluating the resulting SNRs depend on these variances.

   According to the LS method for regression analysis via Eq. (7), the variance of $\hat{a}$ is given by

B1 $$\begin{aligned} \sigma _{\hat{a}}^2=\frac{\sigma _{e_1}^2}{N\sigma _{{\hat{\xi }}}^2}, \end{aligned}$$

where *N* is the sample size, $\sigma _{{\hat{\xi }}}^2=\textrm{Var}({\hat{\xi }})$ is given by Eq. (8), and $\sigma _{e_1}^2$ is the variance of the error term $e_1$ in Eq. (7). It follows from LS regression that $\sigma _{e_1}^2$ equals the normal-distribution-based conditional variance of ${\hat{\eta }}_1$ given ${\hat{\xi }}$. Accordingly,

B2 $$\begin{aligned} \sigma _{e_1}^2=\textrm{Var}({\hat{\eta }}_1)-[\textrm{Cov}({\hat{\eta }}_1,{\hat{\xi }})]^2/\textrm{Var}({\hat{\xi }}) =\sigma _{\eta _1}^2/\rho _{{\hat{\eta }}_1}-\rho _{{\hat{\xi }}}\gamma _1^2\sigma _{\xi }^2, \end{aligned}$$

where the second equal sign follows from the results in Eqs. (8) and (9). The variance $\omega _{aw}^2=\textrm{Var}(\sqrt{N}\hat{a})$ in Eq. (13) is obtained by combining the results in Eqs. (B1), (B2) and (8). Consequently, the squared SNR for the LS estimate $\hat{a}$ is

B3 $$\begin{aligned} \tau _{aw}^2 =\frac{\gamma _1^2}{\gamma _1^2[1/(\rho _{{\hat{\xi }}}\rho _{{\hat{\eta }}_1})-1]+\sigma _{\zeta _1} ^2/(\rho _{{\hat{\xi }}}\rho _{{\hat{\eta }}_1}\sigma _{\xi }^2)}. \end{aligned}$$

When $$\rho _{{\hat{\xi }}}=\rho _{{\hat{\eta }}_1}=1$$, $$\omega _{aw}^2=\sigma _{\zeta _1}^2/\sigma _{\xi }^2$$, and $$\tau _{aw}^2=\gamma _1^2\sigma _{\xi }^2/\sigma _{\zeta _1}^2$$.

   We next obtain the formula for the standard deviations of $\hat{b}$ and $\hat{c}$. According to the LS method, the variance of $e_2$ in Eq. (7) equals the normal-distribution-based conditional variance of ${\hat{\eta }}_2$ given $({\hat{\eta }}_1, {\hat{\xi }})$. That is

B4 $$\begin{aligned} \sigma _{e_2}^2=\sigma _{{\hat{\eta }}_2}^2- \left( \begin{array}{cc} \sigma _{{\hat{\eta }}_2{\hat{\eta }}_1}&\sigma _{{\hat{\eta }}_2{\hat{\xi }}} \end{array} \right) \left( \begin{array}{cc} \sigma _{{\hat{\eta }}_1}^2 &{} \sigma _{{\hat{\eta }}_1{\hat{\xi }}}\\ \sigma _{{\hat{\xi {\hat{\eta }}}}_1}^2 &{}\sigma _{{\hat{\xi }}}^2 \end{array} \right) ^{-1} \left( \begin{array}{c} \sigma _{{\hat{\eta }}_2{\hat{\eta }}_1}\\ \sigma _{{\hat{\eta }}_2{\hat{\xi }}} \end{array} \right) . \end{aligned}$$

Using the results in Eqs. (8), (9) and the textbook formula for the inverse of a $2\times 2$ matrix, we have

B5 $$\begin{aligned} \left( \begin{array}{cc} \sigma _{{\hat{\eta }}_1}^2 &{} \sigma _{{\hat{\eta }}_1{\hat{\xi }}}\\ \sigma _{{\hat{\xi {\hat{\eta }}}}_1}^2 &{} \sigma _{{\hat{\xi }}}^2 \end{array} \right) ^{-1} =\frac{1}{\sigma _{\xi }^2\sigma _{\eta _1}^2/(\rho _{{\hat{\xi }}}\rho _{{\hat{\eta }}_1})-\gamma _1^2\sigma _{\xi }^4} \left( \begin{array}{cc} \sigma _{\xi }^2/\rho _{{\hat{\xi }}} &{} -\gamma _1\sigma _{\xi }^2\\ -\gamma _1\sigma _{\xi }^2 &{} \sigma _{\eta _1}^2/\rho _{{\hat{\eta }}_1} \end{array} \right) . \end{aligned}$$

Combining Eqs. (B4) and (B5), we can rewrite $\sigma _{e_2}^2$ as

B6 $$\begin{aligned} \begin{array}{ll} \sigma _{e_2}^2&{}=\displaystyle \sigma _{\eta _2}^2/\rho _{{\hat{\eta }}_2} -\frac{1}{\sigma _{\xi }^2\sigma _{\eta _1}^2/(\rho _{{\hat{\xi }}}\rho _{{\hat{\eta }}_1})-\gamma _1^2\sigma _{\xi }^4} \left\{ (\beta _1\sigma _{\eta _1}^2 +\gamma _1\gamma _2\sigma _{\xi }^2)^2\sigma _{\xi }^2/\rho _{{\hat{\xi }}}\right. \\ &{}\quad \left. -2\gamma _1(\beta _1\gamma _1+\gamma _2)(\beta _1\sigma _{\eta _1}^2 +\gamma _1\gamma _2\sigma _{\xi }^2)\sigma _{\xi }^4 + (\beta _1\gamma _1+\gamma _2)^2\sigma _{\xi }^4\sigma _{\eta _1}^2/\rho _{{\hat{\eta }}_1}\right\} \\ &{}=\displaystyle \sigma _{\eta _2}^2/\rho _{{\hat{\eta }}_2} -\frac{1}{\gamma _1^2\sigma _{\xi }^2[1/(\rho _{{\hat{\xi }}}\rho _{{\hat{\eta }}_1})-1] +\sigma _{\zeta _1}^2/(\rho _{{\hat{\xi }}}\rho _{{\hat{\eta }}_1})} \left\{ (\beta _1\sigma _{\eta _1}^2 +\gamma _1\gamma _2\sigma _{\xi }^2)^2(1/\rho _{{\hat{\xi }}}-1)\right. \\ &{}\quad \left. +\gamma _1^2(\beta _1\gamma _1+\gamma _2)^2\sigma _{\xi }^4(1/\rho _{{\hat{\eta }}_1}-1) +\beta _1^2\sigma _{\zeta _1}^4 +(\beta _1\gamma _1+\gamma _2)^2\sigma _{\xi }^2\sigma _{\zeta _1}^2/\rho _{{\hat{\eta }}_1} \right\} . \end{array} \end{aligned}$$

According to the formula for LS regression, the covariance matrix of $\textbf{h}=\sqrt{N}(\hat{b},\hat{c})'$ is given by

$$\begin{aligned} \begin{array}{ll} \textrm{Cov}_h&{}=\sigma _{e_2}^2 \left( \begin{array}{cc} \sigma _{\eta _1}^2/\rho _{{\hat{\eta }}_1} &{} \gamma _1\sigma _{\xi }^2\\ \gamma _1\sigma _{\xi }^2 &{}\sigma _{\xi }^2/\rho _{{\hat{\xi }}} \end{array} \right) ^{-1}\\ &{}\displaystyle = \frac{\sigma _{e_2}^2}{\sigma _{\xi }^2\sigma _{\eta _1}^2/(\rho _{{\hat{\xi }}}\rho _{{\hat{\eta }}_1}) -\gamma _1^2\sigma _{\xi }^4} \left( \begin{array}{cc} \sigma _{\xi }^2/\rho _{{\hat{\xi }}} &{} -\gamma _1\sigma _{\xi }^2\\ -\gamma _1\sigma _{\xi }^2 &{} \sigma _{\eta _1}^2/\rho _{{\hat{\eta }}_1} \end{array} \right) . \end{array} \end{aligned}$$

Thus,

B7 $$\begin{aligned}{} & {} \omega _{bw}^2=\textrm{Var}(\sqrt{N}\hat{b}) = \frac{\sigma _{e_2}^2}{\sigma _{\eta _1}^2/\rho _{{\hat{\eta }}_1}-\rho _{{\hat{\xi }}}\gamma _1^2\sigma _{\xi }^2}, \end{aligned}$$

B8 $$\begin{aligned}{} & {} \omega _{cw}^2=\textrm{Var}(\sqrt{N}\hat{c}) = \frac{\sigma _{e_2}^2\sigma _{\eta _1}^2}{\sigma _{\xi }^2\sigma _{\eta _1}^2/\rho _{{\hat{\xi }}} -\rho _{{\hat{\eta }}_1}\gamma _1^2\sigma _{\xi }^4}, \end{aligned}$$

and

$$\begin{aligned} \omega _{bcw}=\textrm{Cov}(\sqrt{N}\hat{b},\sqrt{N}\hat{c}) =- \frac{\gamma _1\sigma _{e_2}^2}{\sigma _{\eta _1}^2/(\rho _{{\hat{\xi }}}\rho _{{\hat{\eta }}_1}) -\gamma _1^2\sigma _{\xi }^2}. \end{aligned}$$

The squared SNRs for $\hat{b}$ and $\hat{c}$ follow from the results in Eqs. (11), (B7) and (B8), and they are, respectively,

B9 $$\begin{aligned}{} & {} \tau _{bw}^2= \frac{(\sigma _{\eta _1}^2/\rho _{{\hat{\eta }}_1}-\rho _{{\hat{\xi }}}\gamma _1^2\sigma _{\xi }^2) [\gamma _1(\beta _1\gamma _1+\gamma _2)\sigma _{\xi }^2(1/\rho _{{\hat{\xi }}}-1)+\beta _1 \sigma _{\zeta _1}^2/\rho _{{\hat{\xi }}}]^2}{\sigma _{e_2}^2\{\sigma _{\zeta _1}^2/(\rho _{{\hat{\xi }}}\rho _{{\hat{\eta }}_1})+ \gamma _1^2\sigma _{\xi }^2[1/(\rho _{{\hat{\xi }}}\rho _{{\hat{\eta }}_1})-1]\}^2}, \end{aligned}$$

B10 $$\begin{aligned}{} & {} \tau _{cw}^2= \frac{(\sigma _{\xi }^2\sigma _{\eta _1}^2/\rho _{{\hat{\xi }}}-\rho _{{\hat{\eta }}_1}\gamma _1^2\sigma _{\xi }^4) [\gamma _1(\beta _1\sigma _{\eta _1}^2+\gamma _1\gamma _2\sigma _{\xi }^2)(1/\rho _{{\hat{\eta }}_1}-1) +\gamma _2\sigma _{\zeta _1}^2/\rho _{{\hat{\eta }}_1}]^2}{\sigma _{\eta _1}^2\sigma _{e_2}^2\{\sigma _{\zeta _1}^2/(\rho _{{\hat{\xi }}}\rho _{{\hat{\eta }}_1})+ \gamma _1^2\sigma _{\xi }^2[1/(\rho _{{\hat{\xi }}}\rho _{{\hat{\eta }}_1})-1]\}^2}. \end{aligned}$$

It follows from Eq. (14) that the squared SNR for the indirect effect $\hat{a}\hat{b}$ can be computed as

B11 $$\begin{aligned} \tau _{abw}^2=\frac{(ab)^2}{a^2\omega _{wb}^2+b^2\omega _{aw}^2}=(1/\tau _{aw}^2+1/\tau _{bw}^2)^{-1}, \end{aligned}$$

where $\tau _{aw}^2$ and $\tau _{bw}^2$ are given in Eqs. (B3) and (B9), respectively. The result in Eq. (15) is obtained by taking the square root of the expression in (B11).

## Asymptotic Variance of $\hat{a}\hat{b}$ Under Path Analysis Via Bartlett-Factor Scores with Estimated Weights

   In this appendix, we derive a formula of the asymptotic variance of the indirect effect $\hat{a}\hat{b}$ for the model in Eq. (7) when ${\hat{\xi }}$, ${\hat{\eta }}_1$ and ${\hat{\eta }}_2$ are the Bartlett-factor scores (BFS). In particular, we will consider the effect of the sampling errors in the estimated weights ${\hat{\textbf{w}}}_x$, ${\hat{\textbf{w}}}_{y_1}$ and ${\hat{\textbf{w}}}_{y_2}$ used in computing ${\hat{\xi }}$, ${\hat{\eta }}_1$ and ${\hat{\eta }}_2$, respectively. To conform with the terminology of BFS, we reformulate the model in Eq. (2) as a confirmatory factor model with parameters ${\varvec{\theta }}=({\varvec{\lambda }}_x',{\varvec{\lambda }}_{y_1}', {\varvec{\lambda }}_{y_2}', \phi _{\xi \eta _1}, \phi _{\xi \eta _2}, \phi _{\eta _1\eta _2}, {\varvec{\psi }}_x',{\varvec{\psi }}_{y_1}',{\varvec{\psi }}_{y_2}')'$, where ${\varvec{\lambda }}_x$, ${\varvec{\lambda }}_{y_1}$ and ${\varvec{\lambda }}_{y_2}$ are vectors of factor loadings for $\textbf{x}$, $\textbf{y}_1$ and $\textbf{y}_2$, respectively; $\textrm{Var}(\xi )=\textrm{Var}(\eta _1)=\textrm{Var}(\eta _2)=1$, and the $\phi$s are the correlations among the latent variables; ${\varvec{\psi }}_x$, ${\varvec{\psi }}_{y_1}$ and ${\varvec{\psi }}_{y_2}$ are the vectors of the diagonal elements of the error-variance matrices ${\varvec{\Psi }}_x$, ${\varvec{\Psi }}_{y_1}$, and ${\varvec{\Psi }}_{y_2}$, respectively. We use ${\varvec{\Sigma }}={\varvec{\Sigma }}({\varvec{\theta }})$ to represent the covariance structure of the confirmatory factor model. For clarity, we will use ${\varvec{\theta }}_0$ to denote the population value of ${\varvec{\theta }}$ in this appendix.

   Let $\textbf{u}=(\textbf{x}',\textbf{y}_1',\textbf{y}_2')'$ represent the population distribution $N({\varvec{\mu }},{\varvec{\Sigma }})$. Let $\textbf{u}_i$, $i=1$, 2, $\ldots$, *N*, be a random sample of $\textbf{u}$, with $\textbf{S}$ being the sample covariance matrix. Fitting ${\varvec{\Sigma }}({\varvec{\theta }})$ to $\textbf{S}$ using the NML method, the resulting estimate of ${\varvec{\theta }}$ is denoted as $\hat{{\varvec{\theta }}}$. The weights ${\hat{\textbf{w}}}_x$, ${\hat{\textbf{w}}}_{y_1}$ and ${\hat{\textbf{w}}}_{y_2}$ in the formulations of the BFS are functions of $\hat{{\varvec{\theta }}}$, and are given by the form

C1 $$\begin{aligned} {\hat{\textbf{w}}}_z=(\hat{{\varvec{\lambda }}}_z'{\hat{{\varvec{\Psi }}}}_{z}^{-1}\hat{{\varvec{\lambda }}}_z)^{-1}{\hat{{\varvec{\Psi }}}}_{z}^{-1} \hat{{\varvec{\lambda }}}_z, \end{aligned}$$

where $\hat{{\varvec{\lambda }}}_z$ is the vector of the estimated factor loadings and ${\hat{{\varvec{\Psi }}}}_z$ is the matrix of the estimated error variances, with *z* representing the block of indicators $\textbf{x}$, $\textbf{y}_1$ and $\textbf{y}_2$, respectively. The resulting ${\hat{\xi }}={\hat{\textbf{w}}}_x'\textbf{x}$, ${\hat{\eta }}_1={\hat{\textbf{w}}}_{y_1}'\textbf{y}_1$ and ${\hat{\eta }}_2={\hat{\textbf{w}}}_{y_2}'\textbf{y}_2$ are then used in Eq. (7), which is estimated by the LS method. The LS estimates of *a*, *b* and *c* are denoted by $\hat{a}$, $\hat{b}$ and $\hat{c}$, respectively.

   We can also compute the BFSs for $(\xi ,\eta _1,\eta _2)$ jointly. When there is no cross-loading or error covariance in the model, the jointly computed weight vector for each latent variable will be the same as in (C1). We will not consider cross-loadings or error covariances in the current development.

### Asymptotic Expansion of the Estimated Weights

   Let ${\varvec{\sigma }}$ be the vector obtained by stacking the columns in the lower-triangular part of ${\varvec{\Sigma }}$, denoted as ${\varvec{\sigma }}=\textrm{vech}({\varvec{\Sigma }})$. The sample counterpart of ${\varvec{\sigma }}$ is $\textbf{s}=\textrm{vech}(\textbf{S})$. Note that both $\textbf{s}$ and ${\varvec{\sigma }}$ are $p^*\times 1$ vectors, where $p^*=p(p+1)/2$ with $p=p_x+p_{y_1}+p_{y_2}$. Let $\textbf{D}_p$ be the duplication matrix and $\textbf{V}=0.5\textbf{D}_p'({\varvec{\Sigma }}^{-1}\otimes {\varvec{\Sigma }}^{-1})\textbf{D}_p$, where $\otimes$ is the notation for the Kronecker product (see, e.g., Schott, 2017, pp. 315–346). Then it follows from equation (13) of Yuan et al. (2002) that the NML estimate $\hat{{\varvec{\theta }}}$ of ${\varvec{\theta }}$ can be expressed as

C2 $$\begin{aligned} \sqrt{N}(\hat{{\varvec{\theta }}}-{\varvec{\theta }}_0) =(\dot{{\varvec{\sigma }}}'\textbf{V}\dot{{\varvec{\sigma }}})^{-1} \dot{{\varvec{\sigma }}}'\textbf{V}\sqrt{N}(\textbf{s}-{\varvec{\sigma }})+o_p(1), \end{aligned}$$

where $\dot{{\varvec{\sigma }}}$ is the derivative matrix $\dot{{\varvec{\sigma }}}({\varvec{\theta }})=\partial {\varvec{\sigma }}({\varvec{\theta }})/\partial {\varvec{\theta }}'$ evaluated at the population value ${\varvec{\theta }}_0$, and $o_p(1)$ is a term that approaches zero in probability. Note that

C3 $$\begin{aligned} \textbf{S}=\sum _{i=1}^N(\textbf{u}_i-{\bar{\textbf{u}}})(\textbf{u}_i-{\bar{\textbf{u}}})'/(N-1)=\sum _{i=1}^N(\textbf{u}_i-{\varvec{\mu }})(\textbf{u}_i-{\varvec{\mu }})'/N+O_p(1/N), \end{aligned}$$

where ${\varvec{\mu }}=E(\textbf{u}_i)$ and $O_p(1/N)$ is a term such that $NO_p(1/N)=O_p(1)$ is bounded in probability (see chapter 14 of Bishop, Fienberg & Holland, 1975). Since we work with centralized data in both SEM and factor-score regression, we can assume ${\varvec{\mu }}=\textbf{0}$ for simplicity without loss of generality. Combining Eqs. (C2) and (C3), we have

C4 $$\begin{aligned} \sqrt{N}(\hat{{\varvec{\theta }}}-{\varvec{\theta }}_0) =(\dot{{\varvec{\sigma }}}'\textbf{V}\dot{{\varvec{\sigma }}})^{-1} \dot{{\varvec{\sigma }}}'\textbf{V}\frac{1}{\sqrt{N}}\sum _{i=1}^N(\textbf{t}_i-{\varvec{\sigma }})+o_p(1), \end{aligned}$$

where $$\textbf{t}_i=\textrm{vech}(\textbf{u}_i\textbf{u}_i')$$.

   Let $\textbf{w}=(\textbf{w}_x',\textbf{w}_{y_1}',\textbf{w}_{y_2}')'$. We need a counterpart of Eq. (C4) for ${\hat{\textbf{w}}}=({\hat{\textbf{w}}}_x',{\hat{\textbf{w}}}_{y_1}',{\hat{\textbf{w}}}_{y_2}')'$, which are functions of $\hat{{\varvec{\theta }}}$. With Eq. (C1), it follows from the rules of matrix differential that

$$\begin{aligned} d\textbf{w}_z&=-({\varvec{\lambda }}_z'{\varvec{\Psi }}_z^{-1}{\varvec{\lambda }}_z)^{-1}[ 2(d{\varvec{\lambda }}_z'){\varvec{\Psi }}_z^{-1}{\varvec{\lambda }}_z- {\varvec{\lambda }}_z'{\varvec{\Psi }}_z^{-1}(d{\varvec{\Psi }}_z){\varvec{\Psi }}_z^{-1}{\varvec{\lambda }}_z] ({\varvec{\lambda }}_z'{\varvec{\Psi }}_z^{-1}{\varvec{\lambda }}_z)^{-1}{\varvec{\Psi }}_z^{-1}{\varvec{\lambda }}_z\\&\quad +({\varvec{\lambda }}_z'{\varvec{\Psi }}_z^{-1}{\varvec{\lambda }}_z)^{-1}[ -{\varvec{\Psi }}_z^{-1}(d{\varvec{\Psi }}_z){\varvec{\Psi }}_z^{-1}{\varvec{\lambda }}_z +{\varvec{\Psi }}_z^{-1}(d{\varvec{\lambda }}_z)]\\&= [-2(d{\varvec{\lambda }}_z')\textbf{w}_z+{\varvec{\lambda }}_z'{\varvec{\Psi }}_z^{-1}(d{\varvec{\Psi }}_z)\textbf{w}_z] \textbf{w}_z +[-{\varvec{\Psi }}_z^{-1}(d{\varvec{\Psi }}_z)\textbf{w}_z +({\varvec{\lambda }}_z'{\varvec{\Psi }}_z^{-1}{\varvec{\lambda }}_z)^{-1}{\varvec{\Psi }}_z^{-1}(d{\varvec{\lambda }}_z)]\\&= \textbf{w}_z[-2\textbf{w}_z'(d{\varvec{\lambda }}_z)+{\varvec{\lambda }}_z'{\varvec{\Psi }}_z^{-1}(d{\varvec{\Psi }}_z)\textbf{w}_z] +[-{\varvec{\Psi }}_z^{-1}(d{\varvec{\Psi }}_z)\textbf{w}_z +({\varvec{\lambda }}_z'{\varvec{\Psi }}_z^{-1}{\varvec{\lambda }}_z)^{-1}{\varvec{\Psi }}_z^{-1}(d{\varvec{\lambda }}_z)]. \end{aligned}$$

Thus,

$$\begin{aligned} \dot{\textbf{w}}_{z\lambda }=\frac{\partial \textbf{w}_z}{\partial {\varvec{\lambda }}_z'}= -2\textbf{w}_z\textbf{w}_z'+({\varvec{\lambda }}_z'{\varvec{\Psi }}_z^{-1}{\varvec{\lambda }}_z)^{-1}{\varvec{\Psi }}_z^{-1} \end{aligned}$$

and

$$\begin{aligned} \dot{\textbf{w}}_{z\psi }= \frac{\partial \textbf{w}_z}{\partial {\varvec{\psi }}_z'} =\{\textbf{w}_z'\otimes [(\textbf{w}_z{\varvec{\lambda }}_z'-\textbf{I}){\varvec{\Psi }}_z^{-1}]\} \textbf{L}_z, \end{aligned}$$

where $\textbf{L}_z=\partial \textrm{vec}({\varvec{\Psi }}_z)/\partial {\varvec{\psi }}_z'$ with $\textrm{vec}(\cdot )$ being the notation for vectorization (see, e.g., Schott, 2017, p. 324), and $\textbf{I}$ is an identity matrix of size $p_z$ (=$p_x$, $p_{y_1}$ or $p_{y_2}$). Note that Theorem 8.11 of Schott (2017) was used in obtaining the expression for $\dot{\textbf{w}}_{z\psi }$. Thus, with ${\varvec{\theta }}'=({\varvec{\lambda }}_x',{\varvec{\lambda }}_{y_1}', {\varvec{\lambda }}_{y_2}', \phi _{\xi \eta _1}, \phi _{\xi \eta _2}, \phi _{\eta _1\eta _2}, {\varvec{\psi }}_x',{\varvec{\psi }}_{y_1}',{\varvec{\psi }}_{y_2}')$, we have

$$\begin{aligned} \dot{\textbf{w}}({\varvec{\theta }})= \frac{\partial \textbf{w}}{\partial {\varvec{\theta }}'}= \left( \begin{array}{cccccccc} \dot{\textbf{w}}_{x\lambda }&{}\;\textbf{0}_{xy_1}&{}\textbf{0}_{xy_2}&{}\; \textbf{0}_{x3}\; &{}\dot{\textbf{w}}_{x\psi }&{}\;\textbf{0}_{xy_1}&{}\textbf{0}_{xy_2}\\ \textbf{0}_{y_1x}&{}\dot{\textbf{w}}_{y_1\lambda }&{}\;\textbf{0}_{y_1y_2}&{} \textbf{0}_{y_13}\; &{}\textbf{0}_{y_1x}&{}\dot{\textbf{w}}_{y_1\psi }&{}\;\textbf{0}_{y_1y_2}\\ \textbf{0}_{y_2x}&{}\textbf{0}_{y_2y_1}&{}\dot{\textbf{w}}_{y_2\lambda }&{} \textbf{0}_{y_2 3}\; &{}\;\textbf{0}_{y_2x}&{}\textbf{0}_{y_2y_1}&{}\dot{\textbf{w}}_{y_2\psi }\\ \end{array} \right) , \end{aligned}$$

where the boldfaced $\textbf{0}$ are matrices of 0 with dimensions indicated by the subscripts. Note that $\sqrt{N}(\hat{{\varvec{\theta }}}-{\varvec{\theta }}_0)=O_p(1)$. It follows from the mean value theorem that

C5 $$\begin{aligned} \sqrt{N}({\hat{\textbf{w}}}-\textbf{w})=\sqrt{N}[\textbf{w}(\hat{{\varvec{\theta }}})-\textbf{w}({\varvec{\theta }}_0)] =\dot{\textbf{w}}(\bar{{\varvec{\theta }}})\sqrt{N}(\hat{{\varvec{\theta }}}-{\varvec{\theta }}_0) =\dot{\textbf{w}}\sqrt{N}(\hat{{\varvec{\theta }}}-{\varvec{\theta }}_0)+o_p(1), \end{aligned}$$

where $\dot{\textbf{w}}(\bar{{\varvec{\theta }}})$ is a matrix such that each row of $\dot{\textbf{w}}({\varvec{\theta }})$ is evaluated at a vector $\bar{{\varvec{\theta }}}$ that lies between $\hat{{\varvec{\theta }}}$ and ${\varvec{\theta }}_0$, and $\dot{\textbf{w}}=\dot{\textbf{w}}({\varvec{\theta }}_0)$. Combining Eqs. (C4) and (C5) yields

C6 $$\begin{aligned} \sqrt{N}({\hat{\textbf{w}}}-\textbf{w}) =\dot{\textbf{w}}(\dot{{\varvec{\sigma }}}'\textbf{V}\dot{{\varvec{\sigma }}})^{-1} \dot{{\varvec{\sigma }}}'\textbf{V}\frac{1}{\sqrt{N}}\sum _{i=1}^N(\textbf{t}_i-{\varvec{\sigma }})+o_p(1). \end{aligned}$$

Equation (C6) will be used to characterize the asymptotic effect of ${\hat{\textbf{w}}}$ on the estimate $\hat{{\varvec{\beta }}}=(\hat{a},\hat{c},\hat{b})'$ and the corresponding $\hat{a}\hat{b}$ in the following subsections.

### The Asymptotic Covariance Matrix of $\hat{{\varvec{\beta }}}$

   We will obtain the asymptotic variance of $\hat{{\varvec{\beta }}}=(\hat{a},\hat{c},\hat{b})'$ in this subsection. Let

C7 $$\begin{aligned}{} & {} g_{i1}({\varvec{\beta }},\textbf{w}_x,\textbf{w}_{y_1},\textbf{w}_{y_2})=(\textbf{w}_x'\textbf{x}_i)[\textbf{w}_{y_1}'\textbf{y}_{i1}-a(\textbf{w}_x'\textbf{x}_i)], \end{aligned}$$

C8 $$\begin{aligned}{} & {} g_{i2}({\varvec{\beta }},\textbf{w}_x,\textbf{w}_{y_1},\textbf{w}_{y_2}) =(\textbf{w}_x'\textbf{x}_i)[\textbf{w}_{y_2}'\textbf{y}_{i2}-c(\textbf{w}_x'\textbf{x}_i)-b(\textbf{w}_{y_1}'\textbf{y}_{i1})], \end{aligned}$$

C9 $$\begin{aligned}{} & {} g_{i3}({\varvec{\beta }},\textbf{w}_x,\textbf{w}_{y_1},\textbf{w}_{y_2})= (\textbf{w}_{y_1}'\textbf{y}_{i1})[\textbf{w}_{y_2}'\textbf{y}_{i2}-c(\textbf{w}_x'\textbf{x}_i)-b(\textbf{w}_{y_1}'\textbf{y}_{i1})], \end{aligned}$$

and $\textbf{g}_i=(g_{i1},g_{i2}, g_{i3})'$. When ${\hat{\xi }}$, ${\hat{\eta }}_1$ and ${\hat{\eta }}_2$ are Bartlett-factor scores, with

$$\begin{aligned} {\bar{\textbf{g}}}_N({\varvec{\beta }},\textbf{w}_x,\textbf{w}_{y_1},\textbf{w}_{y_2})=\frac{1}{N}\sum _{i=1}^N \textbf{g}_i({\varvec{\beta }},\textbf{w}_x,\textbf{w}_{y_1},\textbf{w}_{y_2}), \end{aligned}$$

the LS estimate $\hat{{\varvec{\beta }}}$ for the model in Eq. (7) satisfies

$$\begin{aligned} {\bar{\textbf{g}}}_N(\hat{{\varvec{\beta }}}, {\hat{\textbf{w}}}_x,{\hat{\textbf{w}}}_{y_1},{\hat{\textbf{w}}}_{y_2})=\textbf{0}, \end{aligned}$$

which is an estimating equation. The theory of estimating equation also implies that

$$\begin{aligned} E[{\bar{\textbf{g}}}_N({\varvec{\beta }}_0, \textbf{w}_x,\textbf{w}_{y_1},\textbf{w}_{y_2})]=\textbf{0}, \end{aligned}$$

where ${\varvec{\beta }}_0=(a,c,b)'$ that are given by $a=(\textbf{w}_x'{\varvec{\Sigma }}_{xx}\textbf{w}_x)^{-1}(\textbf{w}_x'{\varvec{\Sigma }}_{xy_1}\textbf{w}_{y_1})$ and

$$\begin{aligned} \left( \begin{array}{c} c\\ b \end{array} \right) = \left( \begin{array}{cc} \textbf{w}_x'{\varvec{\Sigma }}_{xx}\textbf{w}_x&{}\textbf{w}_x'{\varvec{\Sigma }}_{xy_1}\textbf{w}_{y_1}\\ \textbf{w}_{y_1}'{\varvec{\Sigma }}_{y_1x}\textbf{w}_x&{}\;\textbf{w}_{y_1}'{\varvec{\Sigma }}_{y_1y_1}\textbf{w}_{y_1} \end{array} \right) ^{-1} \left( \begin{array}{c} \textbf{w}_x'{\varvec{\Sigma }}_{xy_2}\textbf{w}_{y_2}\\ \textbf{w}_{y_1}'{\varvec{\Sigma }}_{y_1y_2}\textbf{w}_{y_2} \end{array} \right) . \end{aligned}$$

Because the ${\hat{\textbf{w}}}$s are obtained using the NML estimates of confirmatory factor model, they are consistent for $\textbf{w}=\textbf{w}({\varvec{\theta }}_0)$. The theory of estimating equation also implies that $\hat{{\varvec{\beta }}}$ converges to ${\varvec{\beta }}_0$. We will call ${\varvec{\beta }}_0$ the population value of $\hat{{\varvec{\beta }}}$, which is the same as the population value defined in the main body of this article by assuming that ${\hat{\textbf{w}}}$ equals its population counterpart.

   We will use the technique of estimating equation to obtain the asymptotic covariance matrix of $\sqrt{N}\hat{{\varvec{\beta }}}$, which is further used to obtain the asymptotic variance of $\hat{a}\hat{b}$. Since the interest is in estimating ${\varvec{\beta }}$ and characterizing the distribution of $\hat{{\varvec{\beta }}}$, $\textbf{w}_x$, $\textbf{w}_{y_1}$, and $\textbf{w}_{y_2}$ are called nuisance parameters. A systematic study of estimating equation with nuisance parameters is given by Yuan and Jennrich (2000), and the development in this subsection can be regarded as an application of their development. For this application, we need the derivatives of ${\bar{\textbf{g}}}_N({\varvec{\beta }},\textbf{w}_x,\textbf{w}_{y_1}, \textbf{w}_{y_2})$ with respect to the involved parameters, and they are given by

$$\begin{aligned}{} & {} \textbf{C}_{N\beta }=\frac{\partial {\bar{\textbf{g}}}_N}{\partial {\varvec{\beta }}'} =- \frac{1}{N}\sum _{i=1}^N \left( \begin{array}{ccc} \textbf{w}_x'\textbf{x}_i\textbf{x}_i'\textbf{w}_x &{}0 &{}0\\ 0&{}\textbf{w}_x'\textbf{x}_i\textbf{x}_i'\textbf{w}_x &{}\;\textbf{w}_x'\textbf{x}_i\textbf{y}_{i1}'\textbf{w}_{y_1}\\ 0&{}\textbf{w}_{y_1}'\textbf{y}_{i1}\textbf{x}_i'\textbf{w}_x &{}\;\textbf{w}_{y_1}'\textbf{y}_{i1}\textbf{y}_{i1}'\textbf{w}_{y_1} \end{array} \right) \\{} & {} \quad \textbf{C}_{Nw}=\frac{\partial {\bar{\textbf{g}}}_N}{\partial \textbf{w}'} =\frac{1}{N}\sum _{i=1}^N \left( \begin{array}{ccc} r_{i1}\textbf{x}_i'-a\textbf{w}_x'(\textbf{x}_i\textbf{x}_i') &{} \textbf{w}_x'(\textbf{x}_i\textbf{y}_{i1}') &{}\;\textbf{0}\\ r_{i2}\textbf{x}_i'-c\textbf{w}_x'(\textbf{x}_i\textbf{x}_i') &{}\;-b\textbf{w}_x'(\textbf{x}_i\textbf{y}_{i1}') &{}\; \textbf{w}_x'(\textbf{x}_i\textbf{y}_{i2}')\\ -c\textbf{w}_{y_1}'(\textbf{y}_{i1}\textbf{x}_i')&{}\;r_{i2}\textbf{y}_{i1}'-b\textbf{w}_{y_1}'(\textbf{y}_{i1}\textbf{y}_{i1}')&{} \;\;\textbf{w}_{y_1}'(\textbf{y}_{i1}\textbf{y}_{i2}') \end{array} \right) , \end{aligned}$$

where $r_{i1}=\textbf{w}_{y_1}'\textbf{y}_{i1}-a(\textbf{w}_x'\textbf{x}_i)$, $r_{i2}=\textbf{w}_{y_2}'\textbf{y}_{i2}-c(\textbf{w}_x'\textbf{x}_i)-b(\textbf{w}_{y_1}'\textbf{y}_{i1})$. Note that $E(r_{i1}\textbf{x}_i')=\textbf{0}$, $E(r_{i2}\textbf{x}_i')=\textbf{0}$ and $E(r_{i2}\textbf{y}_{i1}')=\textbf{0}$. It follows from the law of large numbers that

$$\begin{aligned} \textbf{C}_{N\beta }=\textbf{C}_{\beta }+o_p(1),\;\; \textbf{C}_{N w}=\textbf{C}_w+o_p(1), \end{aligned}$$

where

C10 $$\begin{aligned} \textbf{C}_{\beta }=- \left( \begin{array}{ccc} \textbf{w}_x'{\varvec{\Sigma }}_{xx}\textbf{w}_x &{}0 &{}0\\ 0&{}\textbf{w}_x'{\varvec{\Sigma }}_{xx}\textbf{w}_x &{}\;\textbf{w}_x'{\varvec{\Sigma }}_{xy_1}\textbf{w}_{y_1}\\ 0&{}\textbf{w}_{y_1}'{\varvec{\Sigma }}_{y_1x}\textbf{w}_x &{}\;\textbf{w}_{y_1}'{\varvec{\Sigma }}_{y_1y_1}\textbf{w}_{y_1} \end{array} \right) \end{aligned}$$

and

C11 $$\begin{aligned} \textbf{C}_w= \left( \begin{array}{ccc} -a\textbf{w}_x'{\varvec{\Sigma }}_{xx} &{} \textbf{w}_x'{\varvec{\Sigma }}_{xy_1} &{}\;\textbf{0}\\ -c\textbf{w}_x'{\varvec{\Sigma }}_{xx} &{}\;-b\textbf{w}_x'{\varvec{\Sigma }}_{xy_1} &{}\; \textbf{w}_x'{\varvec{\Sigma }}_{xy_2}\\ -c\textbf{w}_{y_1}'{\varvec{\Sigma }}_{y_1x}&{}\;-b\textbf{w}_{y_1}'{\varvec{\Sigma }}_{y_1y_1}&{} \;\;\textbf{w}_{y_1}'{\varvec{\Sigma }}_{y_1y_2} \end{array} \right) . \end{aligned}$$

According to the theory of estimating equation (Yuan and Jennrich, 2000), there exists

C12 $$\begin{aligned} \sqrt{N}(\hat{{\varvec{\beta }}}-{\varvec{\beta }}_0) =-\textbf{C}_{\beta }^{-1} [\sqrt{N}{\bar{\textbf{g}}}_N({\varvec{\beta }}_0,\textbf{w}_x,\textbf{w}_{y_1},\textbf{w}_{y_2})+\textbf{C}_w\sqrt{N}({\hat{\textbf{w}}}-\textbf{w})]+o_p(1). \end{aligned}$$

If follows from Eqs. (C6) to (C12) that, for the LS estimate $\hat{{\varvec{\beta }}}$, we have

C13 $$\begin{aligned} \sqrt{N}(\hat{{\varvec{\beta }}}-{\varvec{\beta }}_0){\mathop {\rightarrow }\limits ^{{\mathcal {L}}}}N(\textbf{0},{\varvec{\Omega }}), \end{aligned}$$

where ${\mathop {\rightarrow }\limits ^{{\mathcal {L}}}}$ is the notation for convergence in distribution and

C14 $$\begin{aligned} {\varvec{\Omega }}= \textbf{C}_{\beta }^{-1}(\textbf{B}_{11}+\textbf{B}_{12}+\textbf{B}_{21}+\textbf{B}_{22}){\textbf{C}_{\beta }^{-1}}', \end{aligned}$$

with

$$\begin{aligned} \textbf{B}_{11}= & {} E\{\textbf{g}_i({\varvec{\beta }}_0,\textbf{w}_x,\textbf{w}_{y_1},\textbf{w}_{y_2})\textbf{g}_i'({\varvec{\beta }}_0,\textbf{w}_x,\textbf{w}_{y_1},\textbf{w}_{y_2})\}, \\ \textbf{B}_{12}= & {} \textbf{B}_{21}'= E\{\textbf{g}_i({\varvec{\beta }}_0,\textbf{w}_x,\textbf{w}_{y_1},\textbf{w}_{y_2}) (\textbf{t}_i-{\varvec{\sigma }})']\textbf{V}\dot{{\varvec{\sigma }}} (\dot{{\varvec{\sigma }}}'\textbf{V}\dot{{\varvec{\sigma }}})^{-1}\dot{\textbf{w}}'\textbf{C}_w', \end{aligned}$$

and

$$\begin{aligned} \textbf{B}_{22}= & {} \textbf{C}_w\dot{\textbf{w}}(\dot{{\varvec{\sigma }}}'\textbf{V}\dot{{\varvec{\sigma }}})^{-1} \dot{{\varvec{\sigma }}}'\textbf{V}E[(\textbf{t}_i-{\varvec{\sigma }})(\textbf{t}_i-{\varvec{\sigma }})']\textbf{V}\dot{{\varvec{\sigma }}} (\dot{{\varvec{\sigma }}}'\textbf{V}\dot{{\varvec{\sigma }}})^{-1}\dot{\textbf{w}}'\textbf{C}_w'. \end{aligned}$$

   We next obtain the analytical forms of $\textbf{B}_{11}$, $\textbf{B}_{12}$ and $\textbf{B}_{22}$. Note that, with normally distributed $\textbf{u}$, there exists (see, e.g., Yuan & Bentler, 1999)

C15 $$\begin{aligned} E[(\textbf{t}_i-{\varvec{\sigma }})(\textbf{t}_i-{\varvec{\sigma }})'] =2\textbf{D}_p^+({\varvec{\Sigma }}\otimes {\varvec{\Sigma }})\textbf{D}_p^{+'}=\textbf{V}^{-1}, \end{aligned}$$

where $\textbf{D}_p^+$ is the generalized inverse of $\textbf{D}_p$. Thus,

C16 $$\begin{aligned} \textbf{B}_{22}= \textbf{C}_w\dot{\textbf{w}}(\dot{{\varvec{\sigma }}}'\textbf{V}\dot{{\varvec{\sigma }}})^{-1}\dot{\textbf{w}}'\textbf{C}_w'. \end{aligned}$$

Equation (C15) can also be used to obtain the analytical forms of $\textbf{B}_{11}$ and $\textbf{B}_{12}$. Note that, with $\textbf{u}_i=(\textbf{x}_i',\textbf{y}_{i1}',\textbf{y}_{i2}')'$, we can write the functions $g_{ij}$ in (C7) to (C9) as

$$\begin{aligned} g_{i1}{} & {} =[(\textbf{w}_x',\textbf{0},\textbf{0})(\textbf{u}_i\textbf{u}_i')(-a\textbf{w}_x', \textbf{w}_{y_1}',\textbf{0})' =\textbf{c}_1'\textrm{vec}(\textbf{u}_i\textbf{u}_i') =\textbf{c}_1'\textbf{D}_p\textbf{t}_i, \\ g_{i2}{} & {} =[(\textbf{w}_x',\textbf{0},\textbf{0})(\textbf{u}_i\textbf{u}_i')(-c\textbf{w}_x', -b\textbf{w}_{y_1}',\textbf{w}_{y_2}')'=\textbf{c}_2'\textrm{vec}(\textbf{u}_i\textbf{u}_i') =\textbf{c}_2'\textbf{D}_p\textbf{t}_i, \\ g_{i3}{} & {} =[(\textbf{0},\textbf{w}_{y_1}',\textbf{0})(\textbf{u}_i\textbf{u}_i')(-c\textbf{w}_x', -b\textbf{w}_{y_1}',\textbf{w}_{y_2}')' =\textbf{c}_3'\textrm{vec}(\textbf{u}_i\textbf{u}_i') =\textbf{c}_3'\textbf{D}_p\textbf{t}_i, \end{aligned}$$

where $\textbf{c}_1'=[(-a\textbf{w}_x', \textbf{w}_{y_1}',\textbf{0})\otimes (\textbf{w}_x',\textbf{0},\textbf{0})]$, $\textbf{c}_2'=[(-c\textbf{w}_x', -b\textbf{w}_{y_1}',\textbf{w}_{y_2}')\otimes (\textbf{w}_x',\textbf{0},\textbf{0})]$ and $\textbf{c}_3'=[(-c\textbf{w}_x', -b\textbf{w}_{y_1}',\textbf{w}_{y_2}')\otimes (\textbf{0},\textbf{w}_{y_1}',\textbf{0})]$; and Theorem 8.11 of Schott (2017) was used for the 2nd equal sign in the above equations. Thus, we have

$$\begin{aligned} \textbf{g}_i({\varvec{\beta }},\textbf{w}_x,\textbf{w}_{y_1},\textbf{w}_{y_2})=\textbf{C}_h\textbf{D}_p\textbf{t}_i= \textbf{C}_h\textbf{D}_p(\textbf{t}_i-{\varvec{\sigma }}), \end{aligned}$$

where $\textbf{C}_h=(\textbf{c}_1,\textbf{c}_2,\textbf{c}_3)'$, and the second equal sign follows from the fact that $E(g_{ij})=0$. Thus,

C17 $$\begin{aligned} \textbf{B}_{11}=E(\textbf{g}_i\textbf{g}_i')=\textbf{C}_h\textbf{D}_pE[(\textbf{t}_i-{\varvec{\sigma }})(\textbf{t}_i-{\varvec{\sigma }})']\textbf{D}_p'\textbf{C}_h' =\textbf{C}_h\textbf{D}_p\textbf{V}^{-1}\textbf{D}_p'\textbf{C}_h', \end{aligned}$$

and

C18 $$\begin{aligned} \textbf{B}_{12}=\textbf{C}_h\textbf{D}_pE[(\textbf{t}_i-{\varvec{\sigma }})(\textbf{t}_i-{\varvec{\sigma }})']\textbf{V}\dot{{\varvec{\sigma }}} (\dot{{\varvec{\sigma }}}'\textbf{V}\dot{{\varvec{\sigma }}})^{-1}\dot{\textbf{w}}'\textbf{C}_w' = \textbf{C}_h\textbf{D}_p\dot{{\varvec{\sigma }}} (\dot{{\varvec{\sigma }}}'\textbf{V}\dot{{\varvec{\sigma }}})^{-1}\dot{\textbf{w}}'\textbf{C}_w'. \end{aligned}$$

The formulas in Eqs. (C10)–(C18) are used to numerically evaluate the population signal-to-noise ratio in Sect. 3 of the article.

### The Asymptotic Variance of $\hat{a}\hat{b}$

   We obtain the asymptotic variance of $\hat{a}\hat{b}$ in this subsection, and the so-called delta method will be used for the purpose (see, e.g., Ferguson, 1996). With the result in (C13) and (C14), let ${\varvec{\Omega }}=(\omega _{jk})$, which is a $3\times 3$ matrix. We have the following result

$$\begin{aligned} \sqrt{N}(\hat{a}\hat{b}-ab) {\mathop {\rightarrow }\limits ^{{\mathcal {L}}}}N(0,\omega _{ab\hat{w}}^2), \end{aligned}$$

where

$$\begin{aligned} \omega _{ab\hat{w}}^2=b^2\omega _{11}+2ab\omega _{13}+a^2\omega _{33}. \end{aligned}$$

The signal-to-noise ratio (SNR) for *ab* under BFS regression with estimated weights is then given by

C19 $$\begin{aligned} \tau _{ab\hat{w}}=ab/\omega _{ab\hat{w}}. \end{aligned}$$

Note that, when ${\hat{\textbf{w}}}=\textbf{w}$, the ${\varvec{\Omega }}$ in equation (C14) is reduced to ${\varvec{\Omega }}=\textbf{C}_{\beta }^{-1}\textbf{B}_{11}{\textbf{C}_{\beta }^{-1}}'$. Then the $\tau _{ab\hat{w}}$ in (C19) becomes identical to the $\tau _{abw}$ in Eq. (15).

## Appendix Comparison of the SNRs by SEM and PAWC with $p_x=p_{y_1}=p_{y_2}=10$

   In Sect. 3 of the article we stated that, as the numbers of indicators increase, PAWC will have more advantages over SEM with respect to the SNRs for parameter estimates. This appendix provides the support for the statement, by considering conditions with 10 indicators for $\xi$, $\eta _1$ and $\eta _2$, respectively (i.e., $p_x=p_{y_1}=p_{y_2}=10$). With $p=30$ variables for the mediation model in Eqs. (2) and (3), the vector ${\varvec{\theta }}$ has 63 base parameters. Parallel to the conditions in Sect. 3, a random sample of size 63 is drawn from the uniform distribution on [0, 1]. By adding 0.2 to each of the 63 numbers, the resulting 63 values are used as the population values of ${\varvec{\theta }}$. By independent replications, $N_c=1000$ sets of population values of ${\varvec{\theta }}$ are obtained. Corresponding to each vector ${\varvec{\theta }}$ of 63 numbers, the population values of the SNRs ($\tau$) for ${\hat{\gamma }}_1$, ${\hat{\gamma }}_2$, ${\hat{\beta }}_1$ and ${\hat{\gamma }}_1{\hat{\beta }}_1$ under SEM are subsequently obtained using the formulas presented in Sect. 2 of the article. Population values of the SNRs for $\hat{a}$, $\hat{c}$, $\hat{b}$ and $\hat{a}\hat{b}$ under PAWC are also obtained by paBFS(*w*) (Bartlett-factor scores with population weights), paBFS ($\hat{w}$) (Bartlett-factor scores that accounts for the sampling errors in the estimated weights), and paEWC (equally weighted composites), respectively (Table D).

**Table 8:** Pairwise comparison of the population signal-to-noise ratios (SNR, $\tau$) by four methods over 1000 conditions ($p_x=10$, $p_{y_1}=10$, $p_{y_2}=10$).

	$a(\gamma _1)$	$c(\gamma _2)$	$b(\beta _1)$	$ab(\gamma _1\beta _1)$
$\tau _{paBFS(w)}>\tau _{SEM}$	1000	1000	1000	955
$\tau _{paBFS(\hat{w})}>\tau _{SEM}$	1000	1000	1000	1000
$\tau _{paEWC}>\tau _{SEM}$	933	1000	1000	805
$\tau _{paBFS(\hat{w})}>\tau _{paBFS(w)}$	1000	761	856	1000
$\tau _{paEWC}>\tau _{paBFS(w)}$	0	171	236	136
$\tau _{paEWC}>\tau _{paBFS(\hat{w})}$	0	152	139	43

SEM = structural equation modeling, paBFS (*w*) = path analysis by Bartlett-factor scores with population weights, paBFS ($\hat{w}$) = path analysis by Bartlett-factor scores with estimated weights, paEWC = path analysis with equally weighted composites.

   Table D displays the results of pairwise comparison of the SNR ($\tau$) values by the four methods for each of the 1000 conditions, parallel to those in Table 2. With paBFS($\hat{w}$) corresponding to greater values of $\tau$ than SEM uniformly across the 1000 conditions on all the four parameters, the results show that PAWC outperforms SEM more frequently at $p_x=p_{y_1}=p_{y_2}=10$ than at $p_x=p_{y_1}=p_{y_2}=3$. For estimating the indirect effect, the method paBFS(*w*) outperforms SEM 955 times out of the 1000 conditions at $p_x=p_{y_1}=p_{y_2}=10$, whereas the number was 894 times in Table 2. Comparing the results of Table D against those of Table 2 indicates that paEWC also gains more advantages over SEM at $p_x=p_{y_1}=p_{y_2}=10$.

   Suppose all the factor loadings in Eq. (2) are held constant (e.g., $\lambda _j=\lambda =0.55$, $j=1$, 2, $\cdots$, *p*) and so are the error variances (e.g., $\psi _j=\psi =.60$, $j=1$, 2, $\cdots$, *p*). It can be shown that, with fixed values of $\gamma _1$, $\gamma _2$, $\beta _1$, $\sigma _{\zeta _1}^2=\textrm{Var}(\zeta _1)$ and $\sigma _{\zeta _2}^2=\textrm{Var}(\zeta _2)$ in Eq. (3), the SNRs for the path coefficients and the indirect effect by all the methods will increase as $p_x$, $p_{y_1}$ and $p_{y_2}$ increase. It can also be shown that the reliabilities of ${\hat{\xi }}$, ${\hat{\eta }}_1$ and ${\hat{\eta }}_2$ under PAWC will approach 1.0 as $p_x$, $p_{y_1}$ and $p_{y_2}$ go to infinity, and PAWC will yield values of SNRs equal to those when the latent variables ($\xi$, $\eta _1$ and $\eta _2$) are literally observed. They are also the maximum possible values of the SNRs for the direct and indirect effects. However, SEM will never be able to achieve such values due to estimating the increasing number of factor loadings and error variances.

## Footnotes

1 Because the mediator is a dependent variable in one regression model and a predictor in another regression model, the technique for mediation analysis is better regarded as path analysis although least-squares regression is used in estimating the path coefficients when the analysis is conducted via manifest variables.

2 The value of.05 is arbitrary, and a smaller value will result in more salient covariates.

3 An indicator is called an anchor if its loading is fixed at 1.0 to identify the scale of a latent variable.
